# Cultured fibroblasts of the Okinawa rail present delayed innate immune response compared to that of chicken

**DOI:** 10.1371/journal.pone.0290436

**Published:** 2023-08-22

**Authors:** Masafumi Katayama, Tomokazu Fukuda, Noriko Kato, Takashi Nagamine, Yumiko Nakaya, Nobuyoshi Nakajima, Manabu Onuma

**Affiliations:** 1 Biodiversity Division, National Institute for Environmental Studies, Tsukuba, Ibaraki, Japan; 2 Graduate School of Science and Engineering, Iwate University, Morioka-city, Japan; 3 Okinawa Wildlife Federation, Uruma, Okinawa, Japan; Chang Gung University, TAIWAN

## Abstract

The Okinawa rail is endemic to Okinawa Island and is categorized as an endangered animal. In this study, we focused on innate immunity because it is the first line of host defense. In particular, signals recognizing foreign RNA (e.g., viruses) are important for host defense because they activate the host immune system. The retinoic acid-inducible gene I (*RIG-I*)-like receptor (RLR) families (*RIG-I*, *MDA5*, and *LGP2*) are sensors that activate innate immunity. Therefore, we analyzed these functions in the Okinawa rail using genomic and cellular analyses of fibroblasts. Fibroblasts can be obtained from dead individuals, allowing these cells to be obtained from dead individuals, which is particularly useful for endangered species. The *MDA5* gene of Okinawa rail was sequenced using the Sanger method following PCR amplification and extraction of the amplified sequence from agarose gel. Additionally, mRNA expression analysis of cultured fibroblasts exposed to poly I:C was done. The *MDA5* gene was found to be a mutated nonfunctional gene in the Okinawa rail. The mRNA expression rates of inflammatory cytokine genes type I *IFN*, and *Mx1* were slower in Okinawa rail than in chicken cultured fibroblasts. Similar to the mRNA expression results, cell number and live cell ratio also slowly decreased in the Okinawa rail compared with chicken cultured fibroblasts, indicating that the innate immune reaction differs between chicken and the Okinawa rail. To the best of our knowledge, this is the first experimental evaluation of the loss of function of the Okinawa rail innate immune genes. In conclusion, our results provide a basis for conservation strategies for the endangered Okinawa rail.

## Introduction

The Okinawa rail (*Hypotaenidia okinawae*) is endemic to northern Okinawa Island [1]. The Okinawa rail is categorized as endangered (EN) in the IUCN Red List because its individual numbers are estimated to be approximately 1500 [2]. The Ministry of Environment of Japan lists traffic accidents, habitat destruction, and negative influences by introduced species as risk factors for the reduction in the number of Okinawa rails. Therefore, the Ministry of Environment of Japan and non-profit organizations (NPO) are trying to reduce the risk of extinction and conserve the Okinawa rail. In addition to these risks, infectious diseases also need to be considered as risk factors that would dramatically reduce the number of Okinawa rails, considering that more than 400,000 non-poultry birds, such as wild birds, have died of infectious avian influenza virus between 2021 and 2022 worldwide [3]. Information on the risk of infectious disease in Okinawa rails would benefit their conservation, but this information is quite limited owing to their endangered status.

The innate immune system plays a critical role in host defense in vertebrates, including avians [4]. Members of the retinoic acid-inducible gene I (*RIG-I*)-like receptor (RLR) families (*RIG-I*, *MDA5*, and *LGP2*) are present in the cytoplasm, and *RIG-I* and *MDA5* are important sensors for recognizing virus [5–8]. The innate and adaptive immune systems are activated by RLR-derived signals; therefore, their recognition function plays a critical role in host defense in avians [4]. In avians, species differences are found in recognition systems of RLR families [4,9,10]. For example, the recognition system of RLR families is different between chickens and ducks: chickens lack the *RIG-I* gene, whereas ducks have this gene [4,9,11]. An immune cell would be the first choice to analyze the RLR function, but the barrier of sampling is high because we basically obtained it from live individuals. In contrast to immune cells, fibroblasts can be obtained from dead individuals, and therefore endangered species-derived fibroblasts can be obtained without sacrificing individuals. RLR genes are expressed in mammals and avian fibroblasts; therefore, many studies have been conducted using fibroblasts to analyze the function of RLR family genes [7,9]. In chickens, duck *RIG-I*-transfected chicken cells (DF-1) can recognize the *RIG-I* ligand and induce the expression of antiviral genes, including chicken *IFN-β* and *MX1*, whereas HPAIV titers are significantly reduced [9,12]. This could contribute to the higher resistance of ducks to HPAIV infections. Recently, Lee et al. reported using targeted knockout of *chMDA5* in chicken DF-1 cells in which the loss of *chMDA5* impaired innate immune responses against RNA ligands [13]. Therefore, *MDA5* is a major sensor for the activation of innate immune responses in chickens. Based on these results, cultured fibroblasts could be useful for analyzing the function of the innate immune system. Thus, we predicted that the host defense response of the Okinawa rail could be evaluated by analyzing the cellular response to the stimulation of RLR family genes in cultured cells.

Here, we compared the RLR function of the Okinawa rail with that of chicken to evaluate the host defense response of the Okinawa rail through the innate immune response in cultured fibroblasts. We accessed the publicly available draft genome sequence of the Okinawa rail to obtain its genomic information. Subsequently, the *MDA5* gene was obtained using the Sanger method after PCR amplification and extraction of the amplified sequence from agarose gel. We further exposed cultured fibroblasts to poly I:C for stimulation of the host RLR [5,9,14,15]. After poly I:C exposure, we analyzed the mRNA expression (of the RLR family and downstream genes), cell growth, live cell ratio, and apoptotic cell ratio in Okinawa rail cells.

## Materials and methods

### Animal cells

Chicken fibroblasts were obtained from muscle tissue in a previous study [16]. These fibroblasts were preserved in a liquid nitrogen tank until use. Embryonic muscle-derived fibroblasts were obtained from a domestic duck purchased from a farm (Amatake-Tanohatamura Co., Inc. Tanohata village, Iwate, Japan). Fibroblast cells from the Okinawa rail and whooper swans were obtained from dead animals, such as those killed by vehicles; thus, approval was not required to obtain these samples.

The details described below exclude the exact sampling locations to protect the animals against poaching. All records are available at the National Institute for Environmental Science (NIES).

A dead Okinawa rail was found on May 27, 2005, by the Okinawa Wildlife Federation, a non-profit organization that focuses on the conservation of wild animals in the Okinawa area of the southwest region of Japan. The organization has permission from the Japanese Ministry of the Environment (MOE) to handle and perform first-aid activities on endangered animals. Dead birds were transferred to the National Institute for Environmental Studies (NIES) the following day, and cultured fibroblasts were obtained from their muscle tissue and skin (NIES ID:74A). In this study, skin-derived fibroblasts were used.

Dead whooper swans were found by residents on March 10, 2018, in Morioka City, Iwate Prefecture. They were collected by the staff of Iwate Prefecture and transferred the following day to the NIES. Cultured fibroblasts were obtained from muscle tissue and skin of dead birds (NIES ID:5137A). Skin-derived fibroblasts were used.

### Cell culture

We used KAv-1 medium to obtain and culture chicken, Okinawa rail, domestic duck, and whooper swan fibroblasts. KAv‐1 is based on α‐MEM and contains 5% FBS (SH30396.03; Cytiva, Marlborough, MA, USA), 5% chicken serum (16110082; Thermo Fisher, Waltham, MA, USA), 0.1% D-glucose (041–00595; FUJIFILM Wako Pure Chemical Industries, Osaka, Japan), 0.4 mM calcium chloride, 10 mM EPPS (348–03192; FUJIFILM Wako Pure Chemical Industries), 0.11% sodium carbonate (199–01585; FUJIFILM Wako Pure Chemical Industries), 55 μM 2-mercaptoethanol (21985–023; Thermo Fisher, Waltham, MA, USA), and 1% penicillin-streptomycin-amphotericin B suspension (×100) (Antibiotic-Antimycotic Solution) (161–23181; FUJIFILM Wako Pure Chemical Industries). Cells were cultured at 37°C in 5% CO_2_.

### Staining of F-actin

Chicken muscle-derived, Okinawa rail muscle-derived, and chicken embryonic fibroblasts were seeded in 12-well cell culture plates for immunological staining. After 48 h of incubation, F-actin staining was performed using Alexa Fluor 568 phalloidin (A12380; Thermo Fisher Scientific) according to the manufacturer’s protocol. The samples were counterstained with Cellstain-DAPI solution (DOJINDO). Chicken embryonic fibroblasts were obtained from a primary culture of chicken embryonic tissue provided by Prof. Atsushi Tajima, Tsukuba University.

### Exposure to poly I:C

After preculture for 48 h, fibroblasts were exposed to 5 μg/mL or 50 μg/mL of poly I:C (polyinosinic-polycytidylic acid sodium salt) (4287/10; R&D Systems, Minneapolis, MN, USA). Poly I:C is a mixture of long and short reagents. During preculture and poly I:C exposure, the cells were cultured in Dulbecco’s Modified Eagle Medium (DMEM; 043–30085; FUJIFILM Wako Pure Chemical Industries) containing 10% FBS (SH30396.03; Cytiva) and 1% penicillin-streptomycin-amphotericin B suspension (×100) (Antibiotic-Antimycotic Solution) (161–23181; FUJIFILM Wako Pure Chemical Industries). Cells were cultured at 37°C in 5% CO_2_.

### RNA extraction and quantitative real-time polymerase chain reaction (PCR)

Total RNA was extracted from the cultured cells using NucleoSpin® RNA (740955.50; MACHEREY-NAGEL, Düren, Germany). After measuring the concentration of total RNA from cultured cells, we synthesized complementary DNA (cDNA) from the total RNA. cDNA was synthesized using the PrimeScript™ RT Reagent Kit with gDNA Eraser (Perfect Real Time) (RR047A; Takara Bio, Shiga, Japan). Quantitative real-time PCR was performed in 12.5 μL reaction mixture containing 2×KOD SYBR® qPCR Mix (QKD-201; TOYOBO, Osaka, Japan), 12.5 ng of cDNA, 0.5 μL of Rox, 0.3 μM of each primer, and DW (added up to 12.5 μL), using the Applied Biosystems 7300 system (Thermo Fisher Scientific). The primer sequences are shown in S1–S4 Tables. The cycling conditions were 98°C for 2 min (initial denature), 98°C for 10 s (denature), 58°C for 10 s (annealing), and 68°C for 32 s (extension at) for 45 cycles. The expression levels of the target genes were normalized with those of *GAPDH*.

We searched the sequence information of chicken, swan, and domestic duck from the NCBI database (https://www.ncbi.nlm.nih.gov/gene/?term=) and used Oligo7 (Molecular Biology Insight, Vondelpark Colorado Springs, CO, USA) to design the primers for real-time PCR. We confirmed that the designed primers did not amplify non-target sequences with primer blast (https://www.ncbi.nlm.nih.gov/tools/primer-blast/). During this search, we used the Refseq mRNA as a database, and selected chicken (taxid:9031), domestic duck (taxid:8839), or black swan (taxid:8868) as an organism. Below 400bp amplificated non-target sequence did not find. We consider that our designed primers specifically amplified the target sequence with real-time PCR because the maximum size of the PCR product is around 150 bp.

In addition to chicken, swan, and domestic duck primers, we designed the primers for real-time PCR of Okinawa rail targets. To design the primers for Okinawa rail, we tried to obtain the *RIG-I*, *MDA5*, *LGP2*, *IL6*, *IL1beta*, *IFN-beta*, *Mx1*, *TLR3*, and *GAPDH* sequences. To obtain those Okinawa rail sequences, we searched the Okinawa rail draft sequence (Gallirallus_okinawae_ver1.0 GenBank assembly [GCA_002003005.1] (https://www.ncbi.nlm.nih.gov/assembly/GCA_002003005.1)) using blastn (https://blast.ncbi.nlm.nih.gov/Blast.cgi?PAGE_TYPE=BlastSearch&PROG_DEF=blastn&BLAST_SPEC=Assembly&UID=19747853). We used the chicken mRNA sequence as a query, except for *RIG-I* because there is no chicken ortholog. Therefore, we used the duck *RIG-I* sequence as the query and designed the primers for the Okinawa rail sequences using Oligo7 (Molecular Biology Insight). We confirmed that the designed primers did not amplify non-target sequences using primer blast (https://www.ncbi.nlm.nih.gov/tools/primer-blast/). However, we could not select the Okinawa rail mRNA and therefore used the Okinawa rail genome database (genomic/547194/GCA_002003005.1). No amplified product without the target sequence was observed below 400 bp. Therefore, our designed primers specifically amplified the target sequence with real-time PCR.

### Sequencing of amplified *MDA5* products from chicken and Okinawa rail

The amplified *MDA5* products from chicken and Okinawa rail were sequenced after real-time PCR. First, we collected the chicken and Okinawa rail total RNA after exposure to 0 μg/mL or 50 μg/mL of poly I:C for 24 h using an RNA extraction kit (NucleoSpin® RNA). Next, we synthesized cDNA using the PrimeScript™ RT Reagent Kit with gDNA Eraser (Perfect Real Time) (RR047A; Takara Bio, Shiga, Japan). For cDNA synthesis, we prepared the reverse transcriptase plus (RT (+)) and reverse transcriptase minus (RT (-)). To amplify the cDNA, we used the KOD SYBR® qPCR Mix; the detailed protocol is described in the above section. After amplification, we performed electrophoresis of the amplified *MDA5* products from chicken and Okinawa rail in a 2% agarose gel. After staining with GelGreen (41005; Biotium, Inc., Fremont, CA), the amplified products of 132 bp (chicken) and 82 bp (Okinawa rail) were extracted because these were the predicted *MDA5* targets. The amplified sequences were extracted with NucleoSpin® Gel and PCR Clean-up (740609.50; MACHERY-NAGEL). After those extractions, we stained extracted sequence using BigDye™ Terminator v3.1 Cycle Sequencing Kit (4337457; ThermoFisher) with forward or reverse primers of MDA5. After staining, the samples were sequenced using the Sanger method. We compared the sequences obtained with chicken and Okinawa rail MDA5 sequences using t-coffee (https://tcoffee.crg.eu).

### Quantitative real-time PCR with fluorescence probe

We designed the primers and probe to analyze the *MDA5* gene expression of chicken and Okinawa rail cultured fibroblasts. To design the primers and probe, we used the common sequence between the chicken and Okinawa rail *MDA5*. We also designed the primers and probe for *GAPDH*. Designed primers and probe information are shown in S5 Table. cDNA was synthesized with the PrimeScript™ RT Reagent Kit with gDNA Eraser (Perfect Real Time). Quantitative real-time PCR was performed using 1× THUNDERBIRD Probe qPCR Mix (QPS-101; TOYOBO), 0.3 μM of each primer, 0.2 μM of probe, and 1× Rox. Fifty cycle of 95°C for 60 s (initial denaturation), 95°C for 15 s (denaturation), and 50°C (*MDA5)* or 55  °C (*GAPDH*) 60 s (annealing and extension) were used. The expression levels of the target genes were normalized to those of *GAPDH*.

### Direct sequencing of Okinawa rail MDA5 using the Sanger method

First, we collected the Okinawa rail total RNA after exposure to 50 μg/mL of poly I:C for 24 h using an RNA extraction kit (NucleoSpin® RNA). Next, we synthesized cDNA using the PrimeScript™ RT Reagent Kit with gDNA Eraser (Perfect Real Time) (RR047A; Takara Bio, Shiga, Japan). We designed the primers using the candidate sequence of Okinawa rail *MDA5* genes from the draft genome (S3 Fig) to amplify the *MDA5* gene. Designed-primer information is shown in S6 Table. To amplify the Okinawa rail cDNA of *MDA5*, we used the PrimeSTAR® Max DNA Polymerase (R045A; Takara Bio, Shiga, Japan) or KOD FX Neo (KFX-201; TOYOBO, Osaka, Japan). While using the PrimeSTAR, the first shot PCR was performed in 0.5-μL template cDNA, 12.5-μL reaction mixture containing 2×PrimeSTAR Max Premix, 0.3-μM of each primer, and DW (added up to 25 μL). The cycling conditions were 94°C for 2 min (initial denaturation), 98°C for 10 s (denaturation), 56°C for 10 s (annealing), and 72°C for 60 s (extension) for 45 cycles. Next, the nested PCR was performed in 5 μL of diluted first shot PCR product (which was 1 μL PCR product diluted with 49 μL DW), 12.5 μL of reaction mixture containing 2×PrimeSTAR Max Premix, 0.3 μM of each primer, and DW (added to make up the volume to 25 μL). The cycling conditions were 94°C for 2 min (initial denature), 98°C for 10 s (denature), 56°C for 10 s (annealing), and 72°C for 60 s (extension) for 45 cycles. While using the KOD FX Neo, the first shot PCR was performed in 4-μL template cDNA, 12.5-μL reaction mixture containing 2×PCR buffer for KOD FX Neo, 0.3-μM of each primer, 0.4-mM dNTPs, and DW (added up to 25 μL). The cycling conditions were 94°C for 2 min (initial denaturation), 98°C for 10 s (denaturation), 51°C for 30 s (annealing), and 68°C for 75 s (extension) for 50 cycles. Next, the nested PCR was performed in 5 μL of diluted first shot PCR product (which was 1 μL PCR product diluted with 49 μL DW), 12.5 μL of reaction mixture containing 2×PCR buffer for KOD FX Neo, 0.3 μM of each primer, 0.4 mM of dNTPs, and DW (added up to make up the volume to 25 μL). The cycling conditions were 94°C for 2 min (initial denature), 98°C for 10 s (denature), 56°C for 30 s (annealing), and 68°C for 60 s (extension) for 45 cycles.

We performed electrophoresis of the amplified *MDA5* products from Okinawa rail in a 2-% agarose gel. After staining with GelGreen (41005; Biotium, Inc., Fremont, CA), the amplified products were extracted. The amplified sequences were extracted with NucleoSpin® Gel and PCR Clean-up (740609.50; MACHERY-NAGEL). Thereafter, we stained the extracted sequence using BigDye™ Terminator v3.1 Cycle Sequencing Kit (4337457; ThermoFisher) with forward or reverse primers of *MDA5*. After staining, the samples were sequenced using the Sanger method. We compared the sequences obtained with chicken and Okinawa rail MDA5 sequences using Blast (https://blast.ncbi.nlm.nih.gov/Blast.cgi) and t-coffee (https://tcoffee.crg.eu).

### Analysis of apoptosis

The chicken and Okinawa rail cells were trypsinized and collected after 48 h of poly I:C exposure. The collected cells were suspended and stained using the Muse® Annexin V & Dead Cell Kit (MCH100105; Luminex Corporation, Austin, Texas, USA). The stained cells were analyzed using a Muse Cell Analyzer (0500–3115, Luminex Corporation, Austin, Texas, USA).

### Genomic information

To obtain target Okinawa rail genes (such as *RIG-I* and *LGP2*), we searched the Okinawa rail draft sequence (Gallirallus_okinawae_ver1.0 GenBank assembly [GCA_002003005.1] (https://www.ncbi.nlm.nih.gov/assembly/GCA_002003005.1)) using blastn (https://blast.ncbi.nlm.nih.gov/Blast.cgi?PAGE_TYPE=BlastSearch&PROG_DEF=blastn&BLAST_SPEC=Assembly&UID=19747853). We used the chicken mRNA sequence as query, except for *RIG-I*, because the *RIG-I* ortholog is absent in chicken. Therefore, we used the duck *RIG-I* sequence as query.

### Statistical analysis

First, we tested the normality of our dataset using the chi-square test for goodness of fit. Some data did not show a normal distribution (S7 Table). Therefore, we employed a unified non-parametric analysis for this study as it is not contingent on normal distribution of the data. To compare the three groups, we used the steal-Dwass test, which is a non-parametric version of the Tukey-Kramer test (Figs 3A, 3B, 5C, 7A, 7B, 10A and 10B). As shown in Fig 9C and 9D, the Mann–Whitney U test is used to compare the two groups, which is also a non-parametric analysis. Significant statistical differences are indicated by *(*P*<0.05). We used the statistical analysis software Statcel3 to perform the analyses (Statcel-the Useful Addin Forms on Excel-3rd ed., OMS Publishing, Higashi-Kurume, Tokyo, Japan).

## Results

### Sequence alignment of the RLR family genes (*RIG-I*, *MDA5*, and *LGP2*) between Okinawa rail and chicken or duck

**Fig 1:**
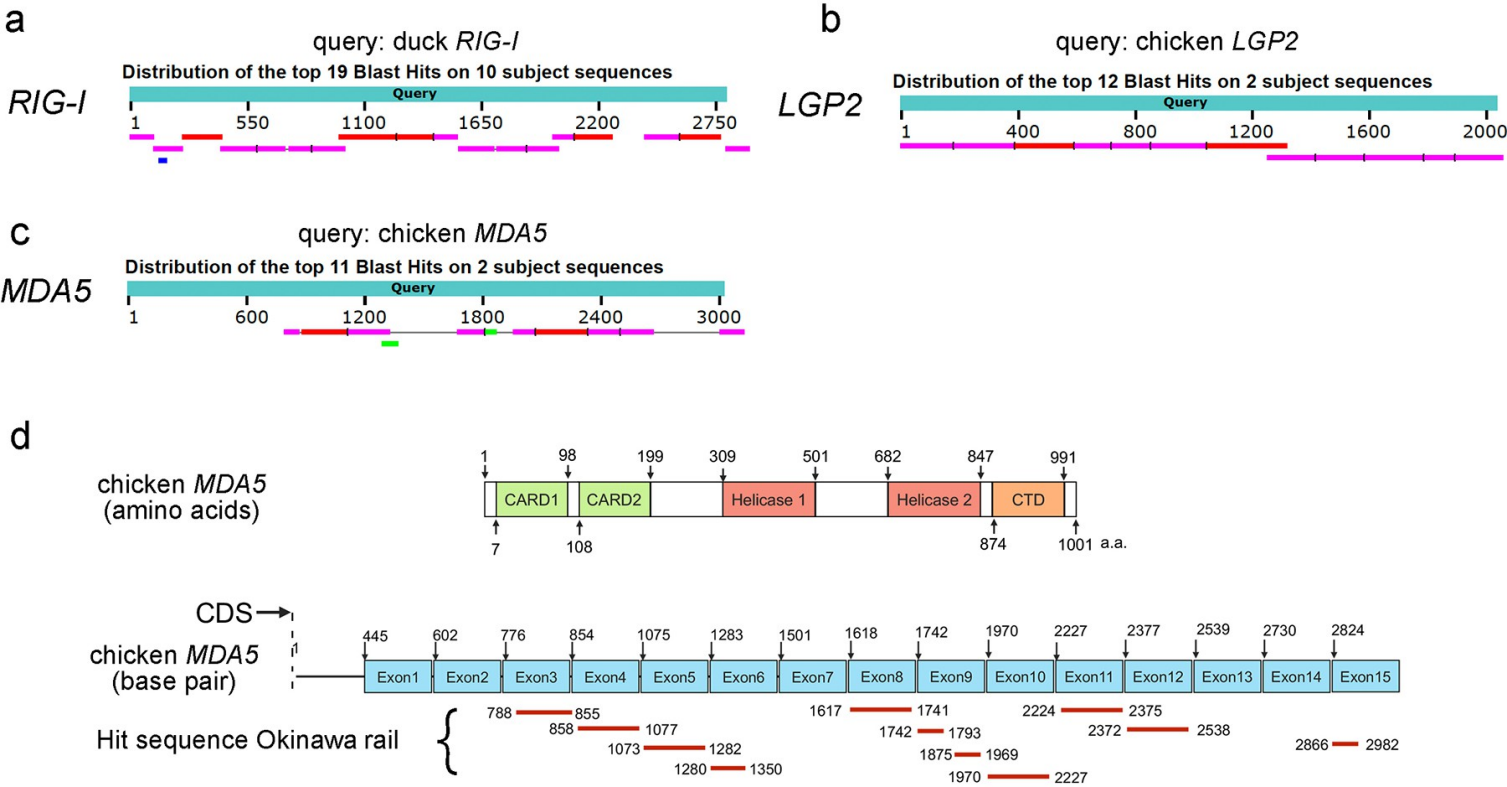
Hit sequences of chicken *LGP2*, *MDA5*, and duck *RIG-I* in Okinawa rail draft genome sequence using blastn. a: Graphical summary of hit sequences of duck *RIG-I* in the Okinawa rail draft genome sequence. b: Graphical summary of hit sequences of chicken *LGP2* in the Okinawa rail draft genome sequence. c: Graphical summary of hit sequences of chicken *MDA5* in the Okinawa rail draft genome sequence. d: Amino acid structure of chicken *MDA5* and mapping of hit sequences of chicken MDA5 in Okinawa rail draft genome sequence to chicken MDA5 coding sequence.

**Fig 2:**
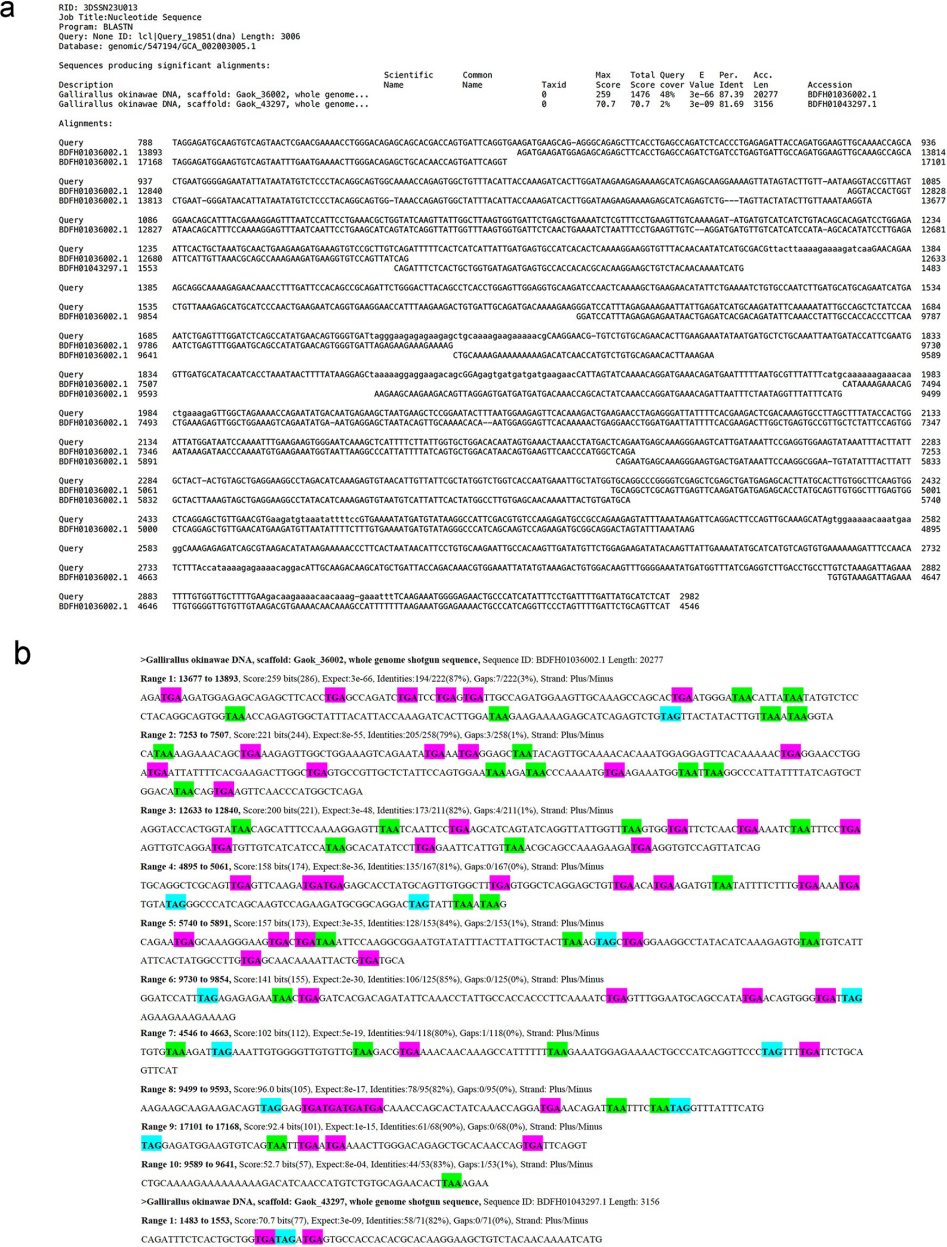
Comparison of the base sequence of chicken *MDA5* and similar sequence in Okinawa rail draft genome sequence. a: Blastn results. The query is chicken *MDA5*. The subject is the Okinawa rail draft genome sequence. b: TGA, TAA, and TAG sequences in the candidate Okinawa rail *MDA5* sequence.

The RLR family recognizes viruses such as influenza. Therefore, we explored the RLR family genes (*RIG-I*, *MDA5*, and *LGP2*) from the draft genome of the Okinawa rail. Our research group has completed the sequencing of the entire genome of the Okinawa rail, and this information has been made available to the public at the DNA databank of Japan (DDBJ), however, gene annotation has not yet been conducted (https://www.ncbi.nlm.nih.gov/genome/?term=okinawa+rail). In this study, we performed a BLAST search for the RLR family mRNA using chicken *LGP2*, chicken *MDA5*, and duck *RIG-I*. We obtained almost full-length sequences of Okinawa rail *RIG-I* and *LGP2* (Fig 1A and 1B), however, hit sequences of the Okinawa rail *MDA5* were partial-length sequences (Fig 1C and 1D). Next, we searched for three sequences (TGA, TAA, and TAG) in the candidate sequence of Okinawa rail *MDA5*, as these sequences could act as stop codons. In the candidate sequence of Okinawa rail *MDA5*, a large number of the three sequences (TGA, TAA, and TAG) were present (TGA: 51, TAA: 33, and TAG: 12) (Fig 2A and 2B). We hypothesized that *MDA5* might be a mutated nonfunctional gene in the Okinawa rail.

### Changes in the RLR family (*RIG-I*, *MDA5*, and *LGP2*) gene expression induced by poly I:C exposure in cultured chicken, Okinawa rail, domestic duck, and whooper swan fibroblasts

**Fig 3:**
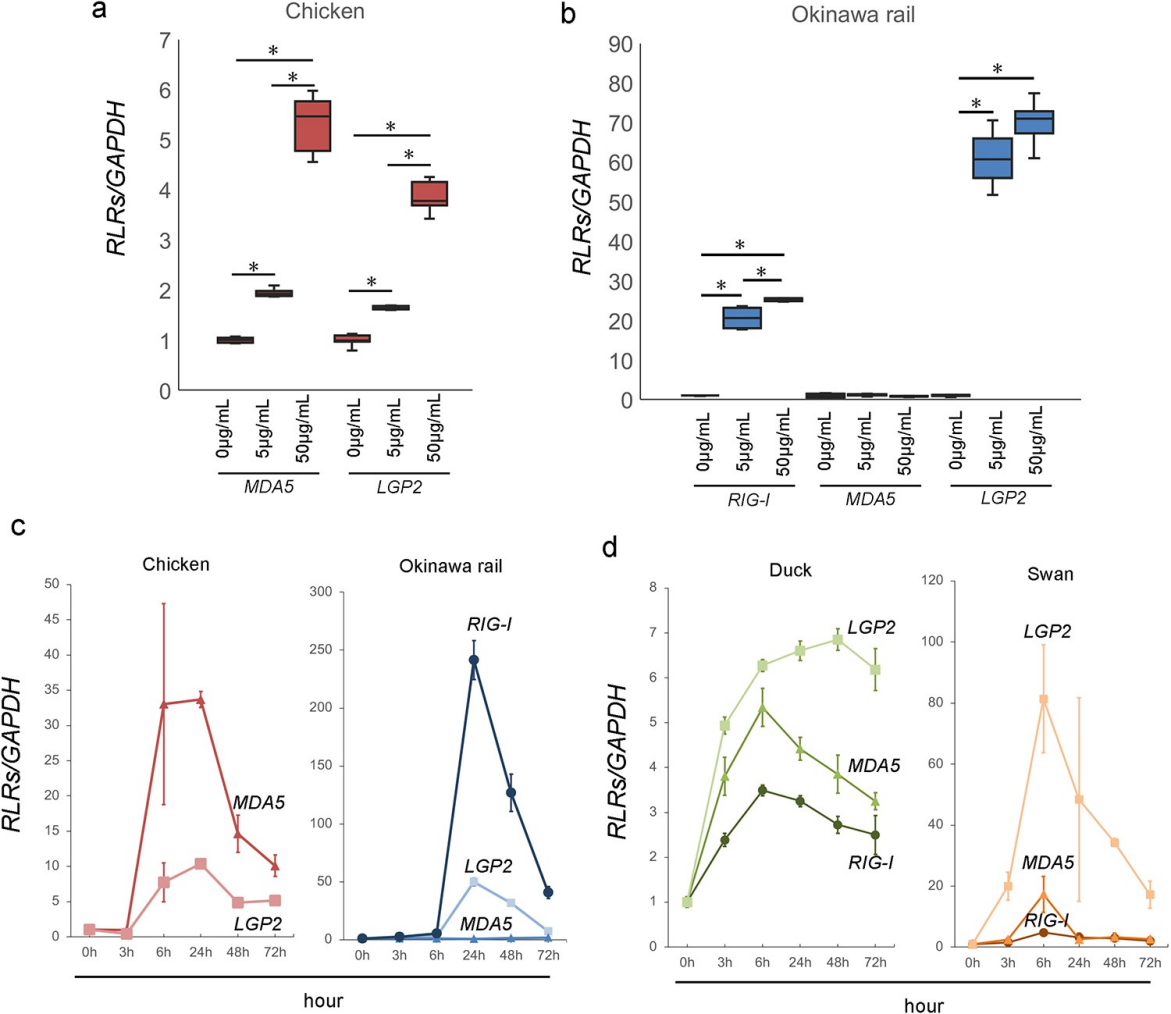
Expression of RLR family genes (*RIG-I*, *MDA5*, and *LGP2*) after poly I:C exposure in chicken and Okinawa rail. a: Expression of *MDA5* and *LGP2* after exposure to 5 μg/mL and 50 μg/mL poly I:C in chicken cultured fibroblasts. Centerlines of box plots indicate medians; box limits indicate 25^th^ and 75^th^ percentiles. Target gene expression was quantified relative to the *GAPDH* internal control. The expression level of the control (poly I:C minus) was 1.0. n = 6. *shows *p* < 0.05. b: Expression of *RIG-I*, *MDA5*, and *LGP2* after exposure to 5 μg/mL and 50 μg/mL poly I:C in Okinawa rail culture cells. Centerlines of box plots indicate medians; box limits indicate 25^th^ and 75^th^ percentiles. Target gene expression was quantified relative to the *GAPDH* internal control. The expression level of the control (poly I:C minus) was 1.0. n = 6. *shows *p* < 0.05. c: Time-course analysis of *RLR* family gene expression after poly I:C exposure in chicken and Okinawa rail cultured fibroblasts. d: Time-course analysis of *RLR* family gene expression after poly I:C exposure in domestic duck and whooper swan cultured cells. Analysis points are 0 (before poly I:C exposure), 3, 6, 24, 48, and 72 h after poly I:C exposure. Error bars show the standard deviation. Target gene expression was quantified relative to the *GAPDH* internal control. Prior to poly I:C exposure, the expression level was 1.0. n = 6.

As shown in Figs 1 and 2, Okinawa rail *MDA5* might be a mutated nonfunctional gene based on the sequence information. To test this hypothesis, we determined the chicken and Okinawa rail RLR family (*RIG-I*, *MDA5*, and *LGP2*) gene expression levels in cultured fibroblasts after stimulation of RLRs. We used chicken muscle-derived and Okinawa rail skin-derived fibroblasts. We first confirmed that the cytoskeleton was similar between chicken muscle-derived and Okinawa rail skin-derived fibroblasts (S1 Fig). Therefore, we considered that the chicken and Okinawa rail cultured fibroblasts had similar characteristics. In chickens, the *RIG-I* gene is not present; therefore, we performed quantitative PCR of *RIG-I* in Okinawa rail [4,9,11]. We analyzed the mRNA expression of the chicken and Okinawa rail RLR family genes after exposure to 5 μg/mL and 50 μg/mL poly I:C for 24 h. In chicken, *MDA5* and *LGP2* mRNA levels were significantly increased with exposure to 5 μg/mL and 50 μg/mL poly I:C compared with 0 μg/mL poly I:C (Fig 3A). In Okinawa rail, *RIG-I* and *LGP2* mRNA levels significantly increased with exposure to 5 μg/mL and 50 μg/mL poly I:C, whereas *MDA5* mRNA expression was not influenced by exposure to poly I:C (Fig 3B). These results indicate that there are species differences in the response of *MDA5* between chicken and Okinawa rail. Particularly, Okinawa rail *MDA5* expression did not increase with stimulation with poly I:C; therefore, this gene lost its function in Okinawa rail during its evolution. This result is consistent with our hypothesis.

In addition to dose-dependent analysis with poly I:C, we also analyzed time-course analysis with poly I:C exposure. In the chicken, *MDA5* and *LGP2* mRNA levels increased after 50 μg/mL poly I:C exposure (Fig 3C). In Okinawa rail, although *RIG-I* and *LGP2* mRNA levels increased after 50 μg/mL poly I:C exposure, *MDA5* mRNA levels remained constant (Fig 3C). The *RIG-I*, *MDA5*, *LGP2* genes are present in duck and swan cells, similar to Okinawa rail. The *RIG-I*, *MDA5*, *LGP2* genes were upregulated in duck and swan cultured fibroblasts after exposure to 50 μg/mL poly I:C (Fig 3D). These results support our hypothesis that Okinawa rail *MDA5* is a mutated nonfunctional gene.

### Sequence of a transcript fragment of *MDA5* in chicken and Okinawa rail

**Fig 4:**
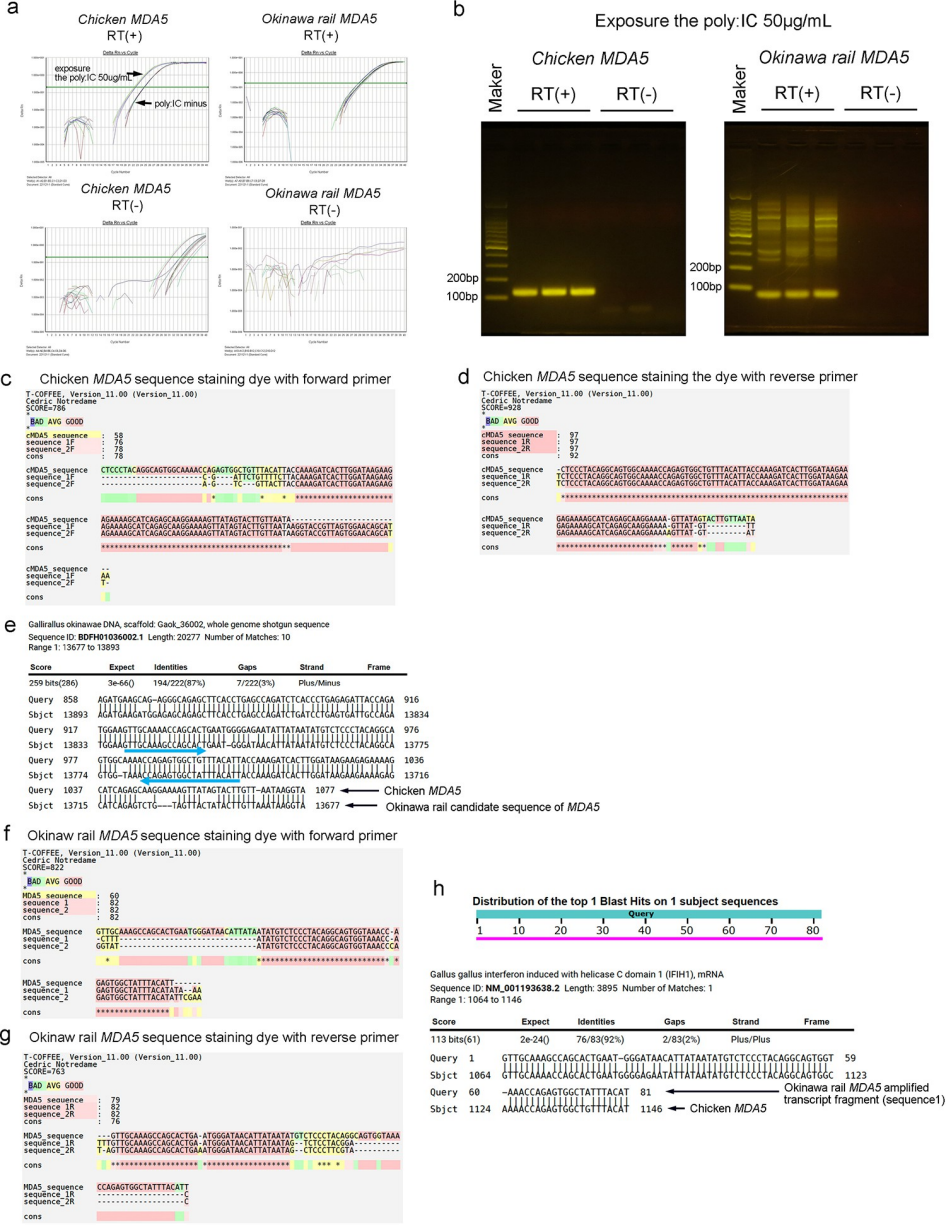
Sequencing of the amplified *MDA5* product from Okinawa rail and chicken using real-time PCR. a: Amplified *MDA5* sequence from chicken and Okinawa rail using real-time PCR staining with SYBR green. Amplification plots of chicken *MDA5* reverse transcriptase plus (RT(+)) (upper left), chicken *MDA5* RT (-) (lower right), Okinawa rail *MDA5* RT (+) (upper light), and Okinawa rail *MDA5* RT (-) (lower light) with real-time PCR. b: Detection of the amplified *MDA5* sequence after real-time PCR. Electrophoretic image of the detection of the amplified *MDA5* sequence from chicken (left panel) and Okinawa rail (right panel). Samples were exposed to 50 μg/mL poly I:C for 24 h. c, d: Comparison between chicken *MDA5* genome sequence and amplified sequence using real-time PCR. The amplified *MDA5* product from chicken was sequenced using the Sanger method. Before sequencing, to stain those sequences with dye, we used the forward or reverse primer of chicken. The stained sequence of the amplified *MDA5* product from chicken was obtained with the forward (c) and reverse (d) primers. e: Primer design of Okinawa rail *MDA5*. Arrows indicate forward and reverse primers of Okinawa rail *MDA5*. f, g: Comparison between Okinawa rail *MDA5* genome sequence and amplified sequence using real-time PCR. Before sequencing, to stain the sequences with dye, we used the forward and reverse primers of Okinawa rail. The stained sequences of the amplified product of *MDA5* transcripts in Okinawa rail were obtained using forward (f) and reverse (g) primers. h: Graphical summary of hit sequences of the amplified product of Okinawa rail *MDA5* in the chicken mRNA database.

In Fig 3, we show *MDA5* mRNA expression staining with SYBR green. We amplified the *MDA5* transcript target in chicken and Okinawa rail using real-time PCR (Fig 4A). We used the two templates to evaluate the origin of the amplified sequence: synthesized cDNA with reverse transcriptase (RT) plus (+) and RT minus (RT(-)) (Fig 4A). We only detected the target sequence of *MDA5* transcript fragments of chicken and Okinawa rail when amplified from synthesized cDNA with RT (+) (Fig 4B). Therefore, the amplified sequences of chicken and Okinawa rail originated from mRNA, not the genome or primer dimers.

We next sequenced the amplified *MDA5* products from chick and Okinawa rail using the Sanger method. For staining the amplified products with dye, we used the forward or reverse primer of chicken and Okinawa rail. The sequence of the amplified *MDA5* product from chicken was highly similar to that of the reference sequence of chicken *MDA5* (Fig 4C and 4D). Therefore, our designed primers amplified the chicken *MDA5* target sequence. We designed the primers for the amplification of Okinawa rail *MDA5* transcripts fragment with the candidate sequence of Okinawa rail *MDA5* (Fig 4E). After sequencing with the Sanger method, our results show that the sequence of the amplified product was highly similar to that of the *MDA5* sequence of Okinawa rail (Fig 4F and 4G). We conclude that our designed primers correctly amplified the Okinawa rail *MDA5* target sequence derived from the candidate sequence of Okinawa rail *MDA5*.

The sequence of the amplified products from Okinawa rail was obtained from both the 5’ and 3’ ends (Fig 4F and 4G). To obtain the full-length sequence of the amplified product from Okinawa rail with real-time PCR, we combined those sequences. Next, we obtained two full-length sequences of the amplified product of Okinawa rail with real-time PCR. Sequence No. 1 was GTTGCAAAGCCAGCACTGAATGGGATAACATTATAATATGTCTCCCTACAGGCAGTGGTAAACCAGAGTGGCTATTTACAT, and sequence No. 2 was GTTGCAAAGCCAGCACTGAAATGGGATAACATTATAATATGTCTCCCTACAGGCAGTGGTAAACCCAGAGTGGCTATTTACAT. After mapping to the chicken RNA reference database, our obtained sequence only hit the *MDA5* mRNA (Fig 4H). Therefore, we considered that our amplified product was identified as the Okinawa rail *MDA5* sequence.

### *MDA5* mRNA expression analysis with fluorescence probe in cultured chicken and Okinawa rail fibroblasts after exposure to poly I:C

**Fig 5:**
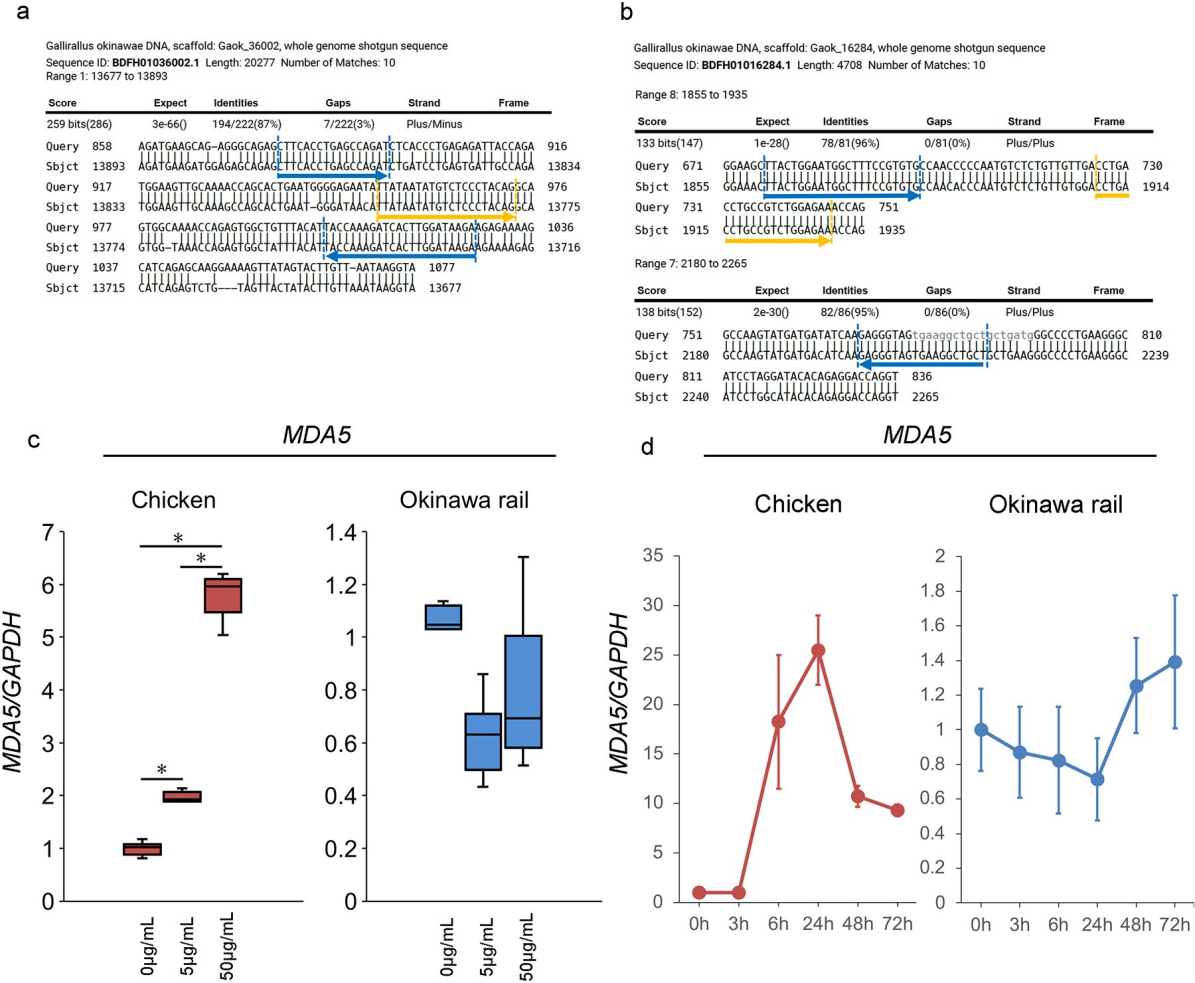
Amplification of chicken and Okinawa rail *MDA5* and *GAPDH* products with fluorescence probe. a, b: Primer and probe design of chicken and Okinawa rail *MDA5* (a) and *GAPDH* (b). Blue arrows show forward and reverse primers. Yellow arrows show fluorescence probes. c: Expression of *MDA5* after exposure to 5 μg/mL and 50 μg/mL poly I:C in the chicken and Okinawa rail cultured fibroblasts. Centerlines of box plots indicate medians; box limits indicate 25th and 75th percentiles. Target gene expression was quantified relative to the *GAPDH* internal control. The expression level of the control (poly I:C minus) was 1.0. n = 6. *shows *p* < 0.05. d: Time-course analysis of *MDA5* expression during poly I:C exposure in chicken and Okinawa rail cultured fibroblasts. The analysis points were 0 (before poly I:C exposure), 3, 6, 24, 48, and 72 h after poly I:C exposure. Error bars show the standard deviation. Target gene expression was quantified relative to the *GAPDH* internal control. Prior to poly I:C exposure, the expression level was 1.0. n = 6.

In addition to mRNA expression analysis of *MDA5* staining with SYBR green, we analyzed *MDA5* mRNA expression with a fluorescence probe. To design the *MDA5* primers and probe, we targeted the common sequences of *MDA5* and *GAPDH* between chicken and the Okinawa rail genomes (Fig 5A and 5B). Similarly to the real-time PCR analysis with SYBR green, even though chicken *MDA5* expression increased with exposure to poly I:C, Okinawa rail *MDA5* expression did not change after exposure (Fig 5C and 5D). Therefore, we considered that Okinawa rail *MDA5* lost its function during its evolution. Based on the results, we regarded that the Okinawa rail *MDA5* gene could be nonfunctional.

### Direct sequencing of *MDA5* of Okinawa rail using the Sanger method

Next, we tried to obtain the long sequence of the ORF of *MDA5* in Okinawa rail. First, we designed the primers based on the candidate sequence of Okinawa rail *MDA5* from the draft genome (S3 Fig). Using these primers, we amplified the *MDA5* sequence from the cDNA of Okinawa rail with PCR (S4A Fig). Additionally, we amplified the sequence with nested PCR (S4A Fig). Next, we performed agarose-gel electrophoresis of the nested-PCR products to visualize the bands (S4B Fig). After the extraction of the amplified sequence from the agarose gel, we read those sequences using the Sanger method.

**Fig 6:**
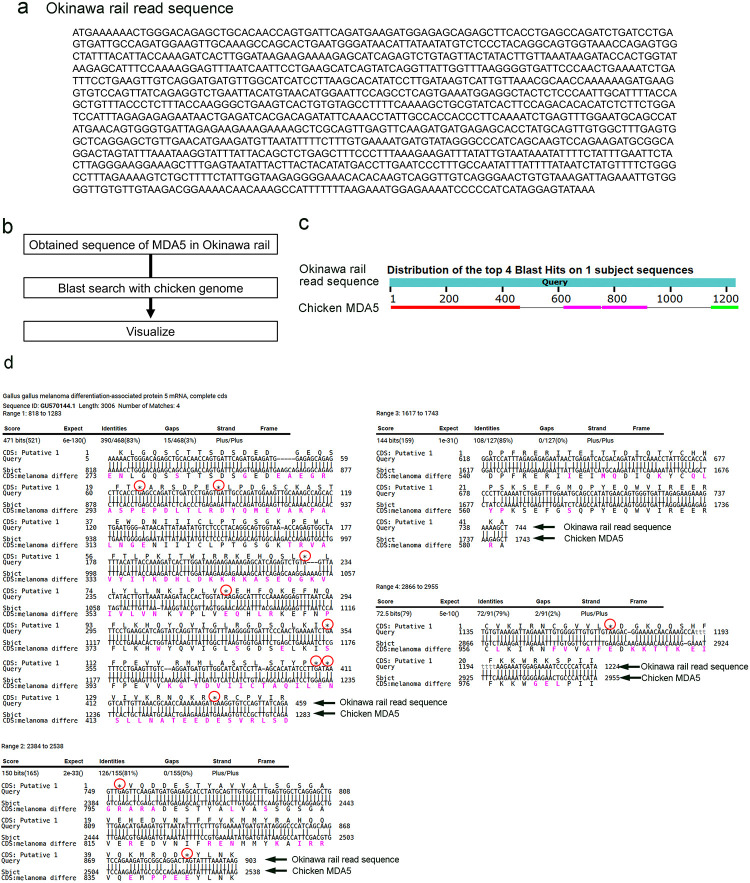
Obtained ORF of the *MDA5* of Okinawa rail. a: Obtained ORF sequence of Okinawa rail *MDA5*. b: blast search flow. c: Graphical summary of the hit sequence of the ORF obtained from Okinawa rail *MDA5* in chicken whole genome information. d: Comparison between the obtained ORF of Okinawa rail and chicken *MDA5*. Red circles denote stop codons.

We obtained the ORF of the *MDA5*, including the ATG start codon, and we found the sequence size to be over 1200 bp (Fig 6A). Next, we performed the Blast search with chicken genome (Fig 6B). These sequences showed homology with the chicken *MDA5* genome (Fig 6C). The Okinawa rail MDA5 amino acid sequence also showed homology with that of the chicken MDA5 amino acid sequence (S4C and S4D Fig). Several stop codons were present in the Okinawa rail *MDA5* sequence (Fig 6D). We observed the deletion of the base in the Okinawa rail *MDA5* sequence (Fig 6D). Therefore, we concluded that Okinawa rail *MDA5* is a non-functional gene, resulting from frameshift mutations. This conclusion was fully consistent with the mRNA expression of Okinawa rail *MDA5*. Thus, we conclude that Okinawa rail *MDA5* gene is nonfunctional.

In Fig 4, the mRNA of Okinawa rail *MDA5* is amplified with real-time PCR. This amplified sequence is contained in our obtained Okinawa rail *MDA5* (S5 Fig). Although we observed the frameshift mutation in this sequence, stop codons were absent in it (S5 Fig). However, we found stop codons upstream of this short sequence. Therefore, we concluded that the levels of Okinawa rail MDA5 mRNA do not increase after exposure to poly I:C.

### Inflammatory cytokine expression induced by poly I:C exposure in cultured chicken, Okinawa rail, domestic duck, and whooper swan cells

**Fig 7:**
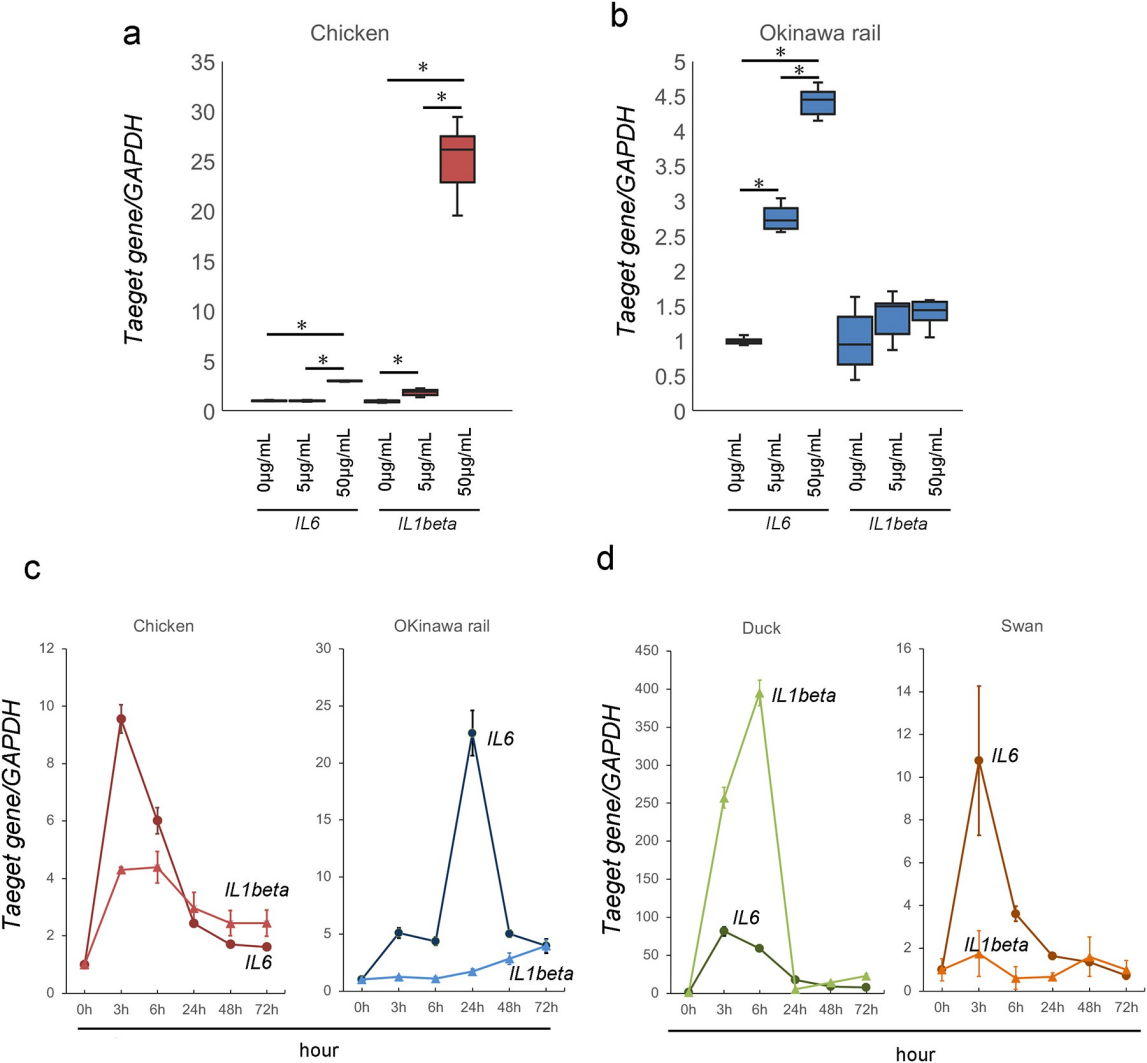
Representative inflammatory cytokine mRNA expression after poly I:C exposure in chicken, Okinawa rail, domestic duck, and whooper swan cells. a: Expression of *IL6* and, *IL1beta* after exposure to 5 μg/mL and 50 μg/mL poly I:C in the chick cultured fibroblasts. Centerlines of box plots indicate medians; box limits indicate 25^th^ and 75^th^ percentiles. Target gene expression was quantified relative to the *GAPDH* internal control. The expression level of the control (poly I:C minus) was 1.0. n = 6. *shows *p* < 0.05. b: Expression of *IL6* and *IL1beta* after exposure to 5 μg/mL and 50 μg/mL poly I:C in the Okinawa rail cultured fibroblasts. Centerlines of box plots indicate medians; box limits indicate 25^th^ and 75^th^ percentiles. Target gene expression was quantified relative to the *GAPDH* internal control. The expression level of the control (poly I:C minus) was 1.0. n = 6. *shows *p* < 0.05. c: Time-course analysis of *IL6* and *IL1beta* expression during poly I:C exposure in chicken and Okinawa rail cultured fibroblasts. d: Time-course analysis of *IL6* and *IL1beta* expression during poly I:C exposure in domestic duck and whooper swan cultured fibroblasts. Analysis points are 0 (before poly I:C exposure), 3, 6, 24, 48, and 72 h after poly I:C exposure. Error bars show the standard deviation. Target gene expression was quantified relative to the *GAPDH* internal control. Prior to poly I:C exposure, the expression level was 1.0. n = 6.

RLR family recognition of poly I:C leads to increased expression of inflammatory cytokines, such as *IL-6* and *IL1-β* [17,18]. We analyzed the mRNA expression of these cytokines from chicken and Okinawa rail after exposure to 5 μg/mL and 50 μg/mL poly I:C for 24 h. In chicken cultured cells, *IL6* and *IL1beta* mRNA levels significantly increased with exposure to 5 μg/mL and 50 μg/mL poly I:C compared with 0 μg/mL poly I:C (Fig 7A). In Okinawa rail cultured cells, *IL-6* mRNA expression levels significantly increased after exposure to poly I:C at 24 h (Fig 7B). Although *IL1-β* mRNA expression levels also increased with exposure to poly I:C for 24 h, the increment was not significant compared to 0 μg/mL poly I:C exposure (Fig 7B). Therefore, the cellular response of inflammatory cytokines to poly I:C exposure is different between chicken and Okinawa rail cell at 24 h.

The maximum expression level of *IL-6* in chicken, domestic duck, and whooper swans was at 3 h but that in Okinawa rail was at 24 h (Fig 7C and 7D). *IL1-β* expression increased in chicken, Okinawa rail, and domestic duck cultured fibroblasts after exposure to 50 μg/mL poly I:C, but swan cell expression levels did not dramatically change (Fig 7C and 7D). Similar to *IL-6* expression, the maximum expression level of *IL1-β* for the Okinawa rail was at a later point compared to that for the chicken (Fig 7B).

These results show that the cellular response of inflammatory cytokines to poly I:C exposure was different between chicken and Okinawa rail cultured fibroblasts. In particular, we considered that these responses were delayed in Okinawa rail cultured fibroblasts compared to that of chicken cultured fibroblasts.

### Cell number and evaluation of live cell ratio in chicken and Okinawa rail cultured fibroblasts after poly I:C exposure

**Fig 8:**
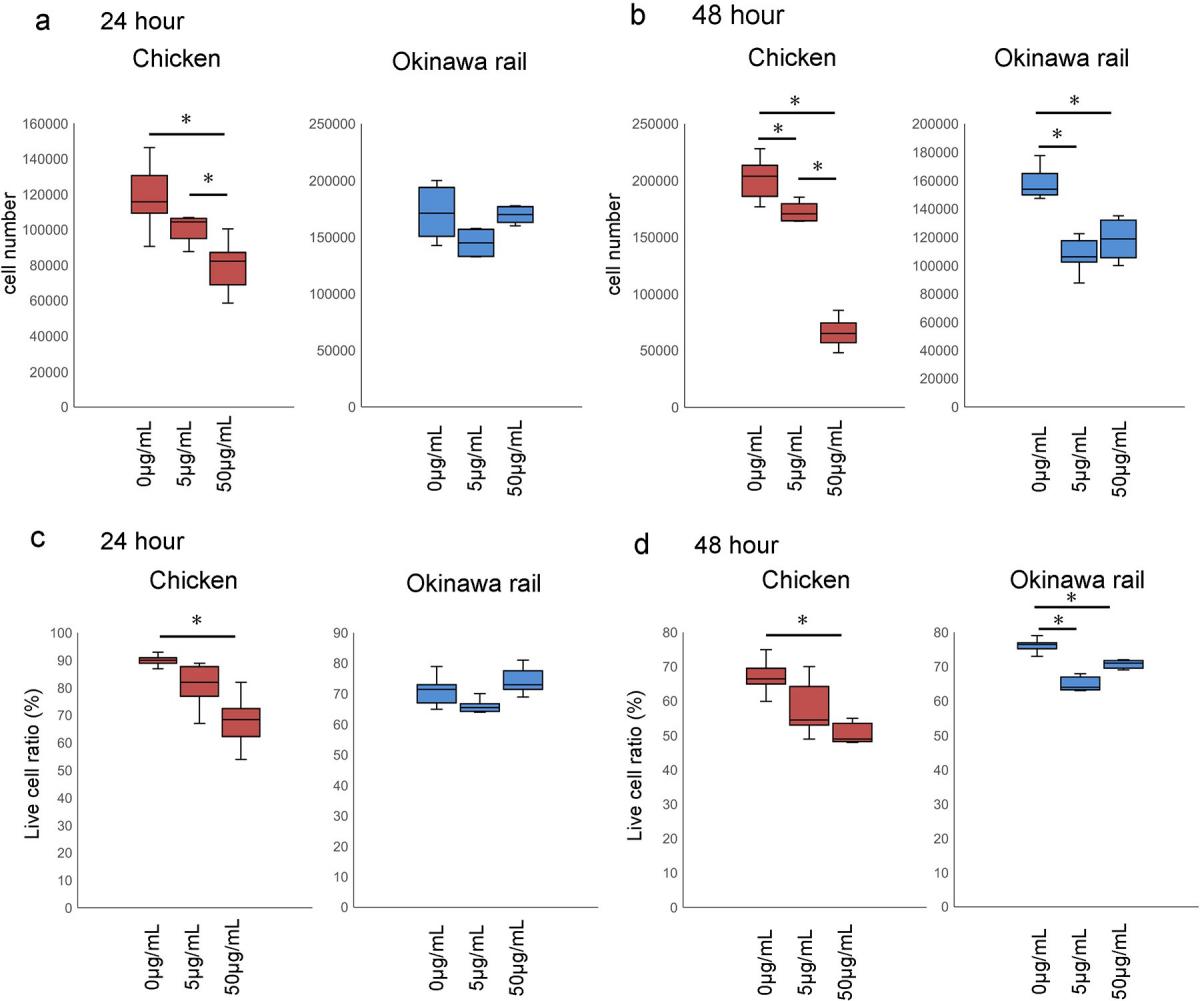
Cell number and live cell ratio of chicken and Okinawa rail cells after poly I:C exposure. a, b: Number of chicken and Okinawa rail cells at 24 h (a) and 48 h (b) after poly I:C exposure. Box plot graphs show the cell numbers of the control (poly I:C minus) and after 5 μg/mL and 50 μg/mL of poly I:C exposure. Centerlines of box plots indicate medians; box limits indicate 25^th^ and 75^th^ percentiles. n = 6. *shows *p* < 0.05. c, d: Live cell ratios of chicken and Okinawa rail cells at 24 h (c) and 48 h (d) after poly I:C exposure. Box plot graphs show the cell numbers of the control (poly I:C minus) and after 5 μg/mL and 50 μg/mL of poly I:C exposure. Centerlines of box plots indicate medians; box limits indicate 25^th^ and 75^th^ percentiles. n = 6. *shows *p* < 0.05.

We evaluated the number of chicken and Okinawa rail cultured fibroblasts after poly I:C exposure for 24 h and 48 h. The number of chicken cultured fibroblasts decreased in a dose-dependent manner at 24 h and 48 h (Fig 8A and 8B). Similar to the cell number, the live cell ratio of chicken cultured fibroblasts also decreased with poly I:C exposure, with a significant decrease after exposure to 50 μg/mL poly I:C (Fig 8C and 8D). In Okinawa rail cultured fibroblasts, the cell number and live cell ratio remained constant at 24 h, regardless of poly I:C exposure (Fig 8A and 8C). Contrastingly, the cell number and live cell ratio of Okinawa rail cells decreased with poly I:C exposure for 48 h (Fig 8B and 8D). The cellular response to poly I:C was different between chicken and Okinawa rail cultured fibroblasts. In particular, the decrease in cell number and live cell ratio was delayed in Okinawa rail cultured fibroblasts compared with that of chicken cultured fibroblasts.

### Apoptosis occurred after poly I:C exposure in chicken cultured fibroblasts

**Fig 9:**
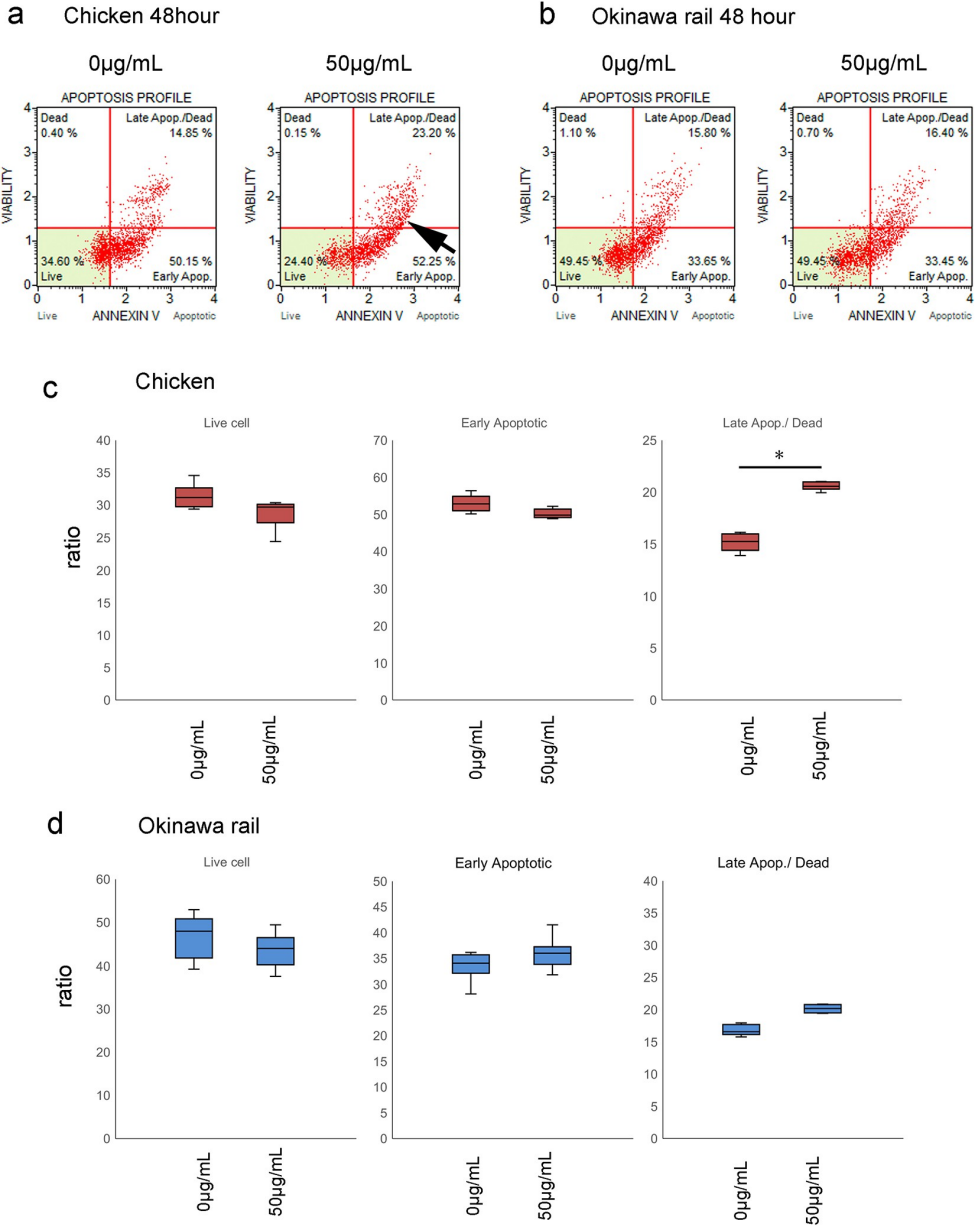
Detection of apoptosis in chicken and Okinawa rail cells after exposure to 50 μg/mL poly I:C. a: Detection of apoptotic cells in chicken after 48 h of exposure to 50 μg/mL poly I:C. b: Detection of apoptotic Okinawa rail cells after 48 h of exposure to 50 μg/mL poly I:C. c, d: Comparison of live cells, early apoptotic cells, and late apoptosis/dead cell ratio between control (poly I:C minus) and 50 μg/mL poly I:C-exposed chicken cells (c) and Okinawa rail cells (d). Left, live cells; middle, early apoptotic cells; right, late apoptosis/dead cells. Box plot graphs show the ratio of control (poly I:C minus) to 50 μg/mL poly I:C-exposed cells. Centerlines of box plots indicate medians; box limits indicate 25^th^ and 75^th^ percentiles. n = 6. *shows *p* < 0.05.

Chicken cultured fibroblasts showed significantly increased late apoptosis and cell death at 48 h (Fig 9A and 9C). In contrast, Okinawa rail cultured fibroblasts did not show a significant change after exposure to 50 μg/mL poly I:C (Fig 9B and 9D). Therefore, exposure to poly I:C for 48 h induced apoptosis in chicken cultured fibroblasts but not in Okinawa rail cultured fibroblasts. These results support our hypothesis that the cellular response to poly I:C exposure differs between chicken and the Okinawa rail.

### *IFN-β* and *Mx1* expression in chicken and Okinawa rail cultured fibroblasts after poly I:C exposure

**Fig 10:**
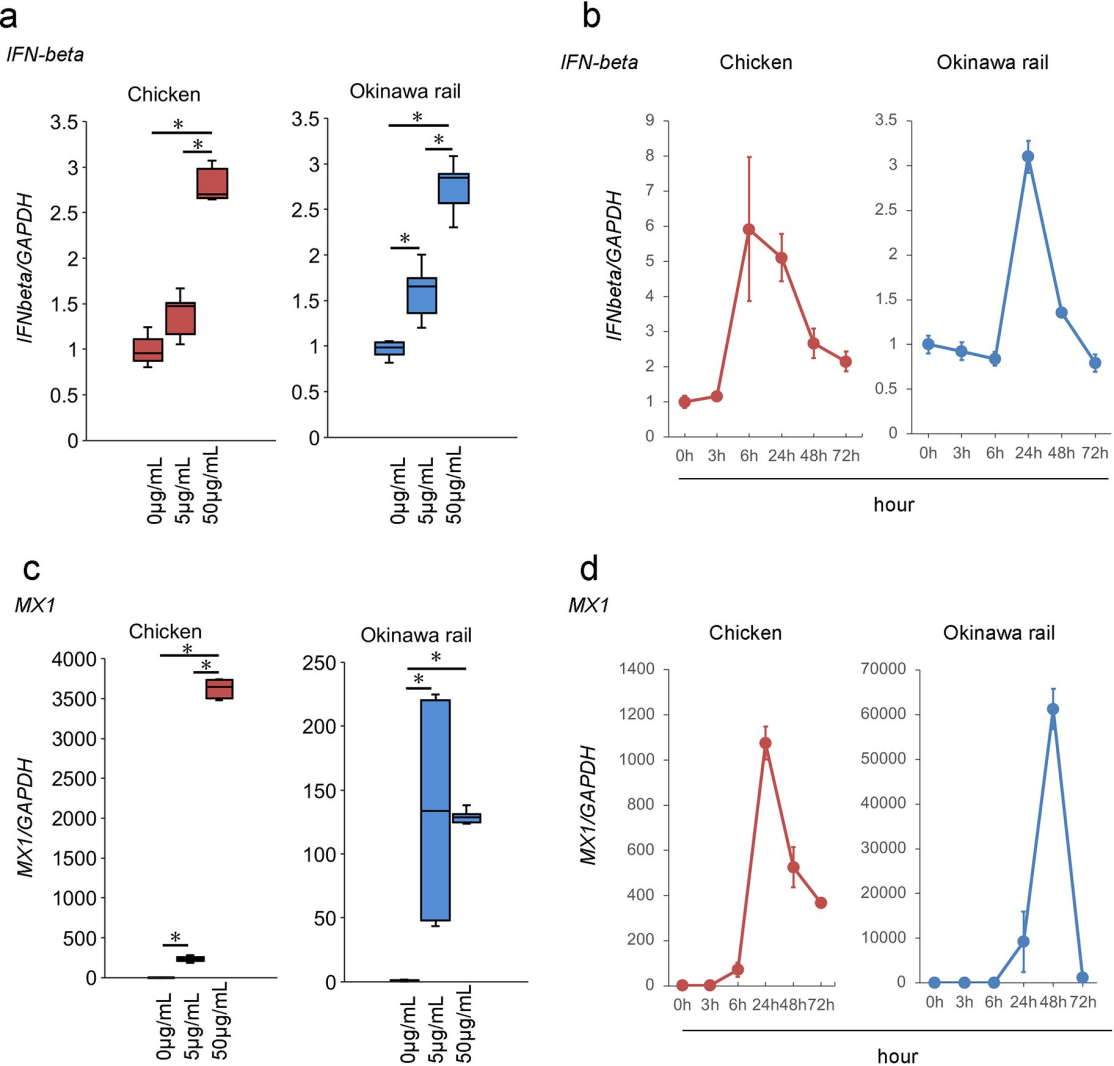
Expression of *IFNβ* and *Mx1* mRNA after poly I:C exposure in chicken and Okinawa rail culture cells. a: Expression of *IFNβ* mRNA after exposure to 5 μg/mL and 50 μg/mL poly I:C (control) in chicken and Okinawa rail cells. Centerlines of box plots indicate medians; box limits indicate 25^th^ and 75^th^ percentiles. *IFNβ* mRNA expression was quantified relative to the *GAPDH* internal control. The expression level of the control (poly I:C minus) was 1.0. n = 6. *shows *p* < 0.05. b: Time-course analysis of *IFNβ* during poly I:C exposure in chicken and Okinawa rail cells. Analysis points are 0 (before poly I:C exposure), 3, 6, 24, 48, and 72 h after poly I:C exposure. Error bars show the standard deviation. *IFNβ* mRNA expression was quantified relative to the *GAPDH* internal control. Prior to poly I:C exposure, the expression level was 1.0. n = 6. *shows *p* < 0.05. c: Expression of *Mx1* mRNA after exposure to 5 μg/mL and 50 μg/mL poly I:C (control) in chicken and Okinawa rail cells. Centerlines of box plots indicate medians; box limits indicate 25^th^ and 75^th^ percentiles. *Mx1* mRNA expression was quantified relative to the *GAPDH* internal control. The expression level of the control (poly I:C minus) was 1.0. n = 6. *shows *p* < 0.05. d: Time-course analysis of *Mx1* mRNA expression during poly I:C exposure in chicken, Okinawa rail, domestic duck, and whooper swan cells. The measurement points are 0 (before poly I:C exposure), 3, 6, 24, 48, and 72 h after poly I:C exposure. Error bars show the standard deviation. Mx1 mRNA expression was quantified relative to the *GAPDH* internal control. Prior to poly I:C, the expression level was 1.0. n = 6. *shows *p* < 0.05.

The *IFN-beta* is activated by *RIG-I-* and *MDA5-* derived signals [4,5]. Our results show that *IFN-β* mRNA expression dose-dependently increased with the amount of poly I:C in chicken and Okinawa rail cultured fibroblasts (Fig 10A). Similar to inflammatory cytokines, the time point of the maximum value of *IFN-β* mRNA expression was delayed in Okinawa rail cultured fibroblasts compared with that in chicken cultured fibroblasts (chicken: 6 h, Okinawa rail: 24 h) (Fig 10B).

Mx1 protein suppresses the intracellular self-renewal of viruses, such as that of the influenza virus [19,20], and the Mx1 protein induces *IFN-β* signaling [19]. Chicken and Okinawa rail cultured fibroblasts showed significantly increased *Mx1* mRNA expression after exposure to 5 μg/mL and 50 μg/mL poly I:C compared with 0 μg/mL poly I:C exposure (Fig 10C). In addition to inflammatory cytokine expression, *IFN-β* and *Mx1* mRNA expression associated with poly I:C exposure was also delayed in Okinawa rail cultured fibroblasts compared with that in chicken cultured fibroblasts (Fig 10D). These results also indicate that Okinawa rail cultured fibroblasts show delayed response to poly I:C compared with that shown by chicken cultured fibroblasts.

## Discussion

RLR families (*RIG-I*, *MDA5*, and *LGP2)* act as sensors for stimulation of the host’s innate immune system [4–8]. Therefore, their recognition function is critical in host defense in birds [4,5]. In avians, the recognition systems of the RLR families differ among species [10], for example, the lack of the *RIG-I* gene in chickens [4,9]. Therefore, we considered that obtaining the sequence of Okinawa rail RLR families would be a clue to their reactivity of host defense to infectious disease. Therefore, we first tried to obtain the RLR family genes (*RIG-I*, *MDA5*, and *LGP2*) from the draft genome of the Okinawa rail. Although the almost full sequence of *RIG-I* and *LGP2* allowed us to obtain the Okinawa rail draft genome with a blastn search, the candidate sequence of *MDA5* was partial. Zheng and Satta reported that avian MDA5 has the highest conservation level in the helicase domain but a lower level in the caspase recruitment domain (CARDs) region using predicted coding sequences from 62 bird species [10]. The corresponding sequence of the CARD region (approximately 1 to 600 base pairs from the N-terminal) was not obtained from the Okinawa rail draft genome sequence in this study; therefore, our results agree with those of previous reports. In the candidate sequence of Okinawa rail *MDA5*, a number of TGA, TAA, and TAG sequences were found; therefore, we considered that stop codons occurred in the Okinawa rail *MDA5* sequence. Based on these results, we suspect that *MDA5* is a mutated nonfunctional gene in the Okinawa rail.

In this study, we analyzed the mRNA expression of *MDA5* in Okinawa rail cultured fibroblasts. Although phagocytosis and antigen presentation occurs only in immune cells (e.g., macrophages and dendritic cells), fibroblasts recognize antigens through RLRs. Therefore, fibroblasts can be used for RLR-derived signaling studies in humans, mice, and chickens [9,15,21]. To stimulate the RLR pathway, we used poly I:C, which has been used in a number of studies for stimulation of the RLR pathway [5,9,14,15]. In the present study, poly I:C stimulated *RIG-I*, *LGP2*, and downstream signals but did not alter *MDA5* mRNA expression in Okinawa rail cultured fibroblasts. In addition to mRNA expression analysis of Okinawa rail *MDA5* after poly I:C exposure, we tried to obtain the ORF of *MDA5*, including the ATG start codon. As consequently, we obtained the ORF of the *MDA5*, including the ATG start codon and the sequence size is over 1200bp. In those sequences, we observed a number of stop codons. This result is fully consistent with our hypothesis. Based on these results, we conclude that *MDA5* of Okinawa rail is a mutated nonfunctional gene. To the best of our knowledge, this is the first experimental evaluation of the loss of function of Okinawa rail genes.

We considered that the loss of function of the *MDA5* gene is critical for the innate immune response of the Okinawa rail. In mammals, *RIG-I* is the main sensor for detecting the influenza A virus, whereas *MDA5* is the primary influenza A virus sensor in chicken [22,23]. *MDA5* recognizes dsRNA of the influenza A virus, resulting in type I *IFN* induction in chicken cells [22]. In the present study, we showed that *IFN-β* mRNA expression was delayed in Okinawa rail after exposure to poly I:C compared to that in chicken. The Mx protein is induced by an *IFN-β* signal and exhibits antiviral activity against RNA viruses, such as the influenza virus [19,20]. In the present study, *Mx1* mRNA expression was delayed in Okinawa rail compared to that in chickens. These results indicate that the innate immune response of the Okinawa rail is delayed when compared to that of chickens. This result is consistent with our hypothesis that the loss of function of the *MDA5* gene has a critical effect on the innate immune response of the Okinawa rail.

Furthermore, we observed that inflammatory cytokine mRNA expression in Okinawa rail was delayed compared with that in chickens, domestic ducks, and whooper swans after poly I:C exposure. Cell number and live cell ratio analysis in chicken cells showed a rapid decrease in cell number and live cell ratio with poly I:C exposure compared with that in Okinawa rail cultured fibroblasts. Late apoptosis/dead cells were only significantly increased in chicken cells after 48 h of poly I:C exposure. Based on these *in vitro* experiments, we conclude that the innate immune response of Okinawa rail cultured fibroblasts was delayed compared with that of chicken cultured fibroblasts.

In addition to RLRs, toll-like receptor (TLR) 3 is stimulated by poly I:C in avian cells [24]. In the present study, we observed that *TLR*3 mRNA expression increased with exposure to poly I:C (S2 Fig). Similar to RLRs, TLRs are sensors for the recognition of antigens, and TLR-derived signals activate innate immunity in birds [4,5]. Although a single knockout of *MDA5* in chicken DF-1 reduced the innate immune responses against RNA ligands, a single knockout of *TLR3* maintained innate immune responses against RNA ligands [13]. Therefore, it has been suggested that *MDA5* is the major sensor, whereas *TLR3* is a secondary sensor in chicken. Therefore, we also considered that RLR in Okinawa rail would be the major sensor for virus recognition compared with *TLR 3*.

The innate immune response plays a critical role in host defense, especially during the early stages. Our *in vitro* study indicated that the *MDA5* gene is a mutated nonfunctional gene in the Okinawa rail and that the innate immune response of the Okinawa rail is delayed compared to that of chicken. Our results provide useful information for the conservation of Okinawa rail because studies of the Okinawa rail immune response are quite limited.

## Supporting information

S1 Fig Comparison of the cytoskeleton of chick muscle-derived fibroblast, Okinawa rail skin derived fibroblast, and chicken embryonic fibroblast. Images show the cytoskeleton of chick muscle-derived fibroblast (upper three panels), Okinawa rail skin-derived fibroblast (middle three panels), and chicken embryonic fibroblast (lower three panels). The left panels show merge images; the middle panels show staining with F-actin; the right panels show the image of Dapi. Scale bar show 100 μm.
(PDF)

S2 Fig Expression of *TLR3* mRNA after poly I:C exposure in chicken and Okinawa rail culture cells. Expression of TLR3 mRNA after exposure to 5 μg/mL and 50 μg/mL poly I:C (control) in chick and Okinawa rail cells. Left side is chicken, right side is Okinawa rail. Centerlines of box plots indicate medians; box limits indicate 25th and 75th percentiles. TLR3 mRNA expression was quantified relative to the GAPDH internal control. The expression level of the control (poly I:C minus) was 1.0. n = 6. *shows p < 0.05.
(PDF)

S3 Fig Primer design for the direct sequencing of Okinawa rail *MDA5*. a: Our designed primer location in the candidate sequence of Okinawa rail MDA5 from the draft genome. b-d: Designed primers of this study.
(PDF)

S4 Fig Amino acids sequence of the obtained Okinawa rail *MDA5*. a: Flow of obtaining the Okinawa rail MDA5. b: Image of electrophoresis after nested PCR. We extracted the amplification sequence from the white arrows. c: Amino acids sequence of our obtained Okinawa rail MDA5. Those sequences were translated from our obtained Okinawa rail MDA5 sequence (Fig 6A). Red asterisks are stop codons. d: Homology between chicken MDA5 and our Okinawa rail MDA5 amino acids sequence.
(PDF)

S5 Fig Translation of the 81 bp cDNA fragment into an amino acid sequence. The orange line is the 81bp cDNA fragment of Fig 4H. The blue highlight sequences are translated amino acids sequences of Okinawa rail that of 81bp cDNA fragment. The green highlight sequences are translated amino acids sequences of chicken that of 81bp cDNA fragment. Asterisks are stop codons.
(PDF)

S1 Table Primer sequence for chicken qPCR. (PDF)

S2 Table Primer sequence for Okinawa rail qPCR. (PDF)

S3 Table Primer sequence for domestic duck qPCR. (PDF)

S4 Table Primer sequence for whooper swan qPCR. (PDF)

S5 Table Primer sequence for qPCR with fluorescence probe. (PDF)

S6 Table Primer sequence for direct sequencing. (PDF)

S7 Table Chi-square test. (PDF)
