# Supplementary figures and images for: Cultured fibroblasts of the Okinawa rail present delayed innate immune response compared to that of chicken
Source: PLoS One. 2023 Aug 22;18(8):e0290436. doi: 10.1371/journal.pone.0290436 (PMC10443837; doi:10.1371/journal.pone.0290436)

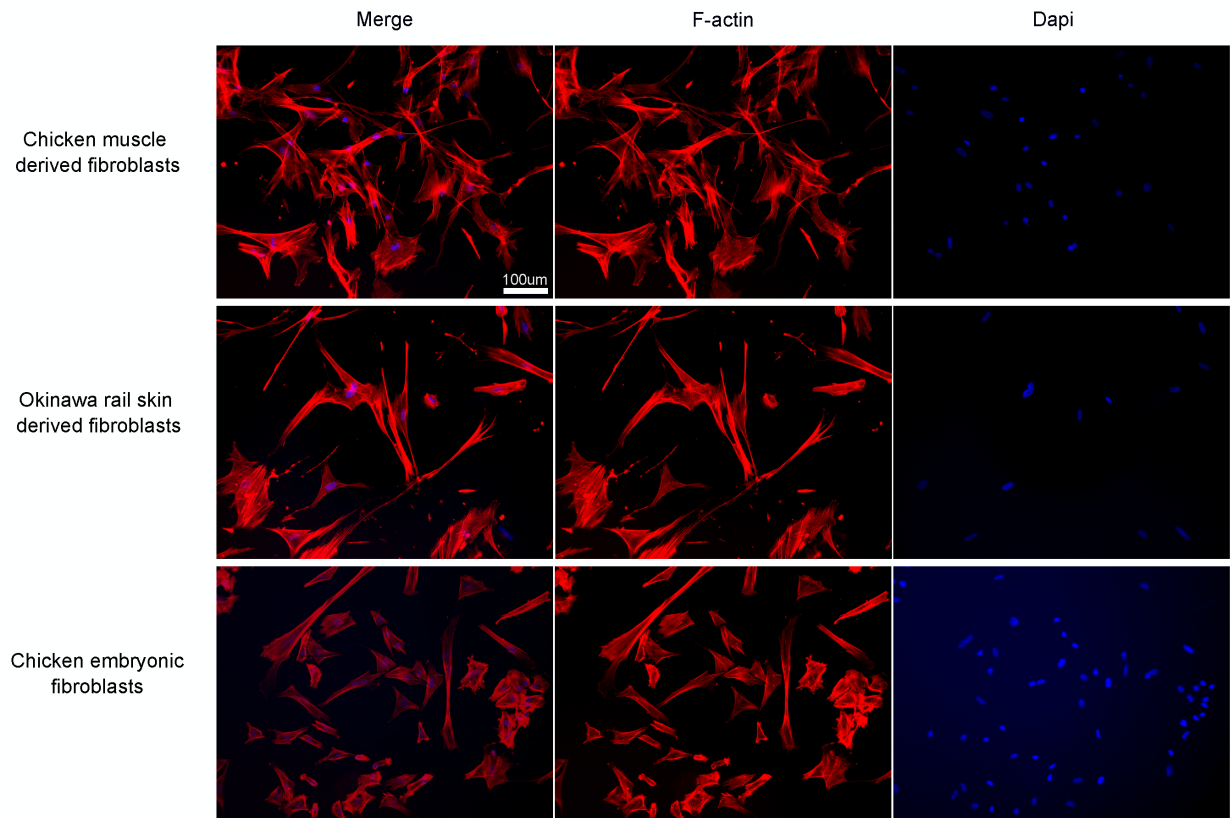

Supplement: S1 Fig — Images show the cytoskeleton of chick muscle-derived fibroblast (upper three panels), Okinawa rail skin-derived fibroblast (middle three panels), and chicken embryonic fibroblast (lower three panels). The left panels show merge images; the middle panels show staining with F-actin; the right panels show the image of Dapi. Scale bar show 100 μm. (PDF) [file pone.0290436.s001.pdf]

TLR3

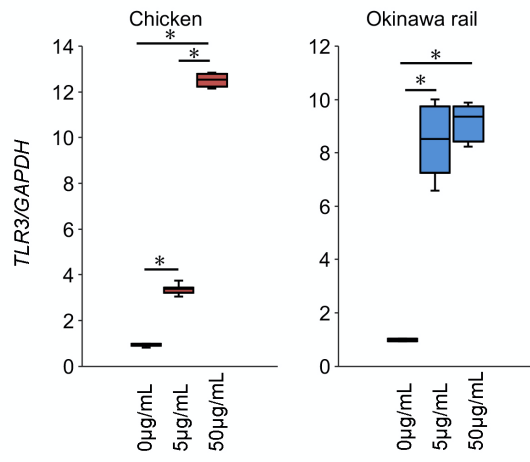

Supplement: S2 Fig — Expression of TLR3 mRNA after exposure to 5 μg/mL and 50 μg/mL poly I:C (control) in chick and Okinawa rail cells. Left side is chicken, right side is Okinawa rail. Centerlines of box plots indicate medians; box limits indicate 25th and 75th percentiles. TLR3 mRNA expression was quantified relative to the GAPDH internal control. The expression level of the control (poly I:C minus) was 1.0. n = 6. *shows p < 0.05. (PDF) [file pone.0290436.s002.pdf]
