# Supplementary material for: Cultured fibroblasts of the Okinawa rail present delayed innate immune response compared to that of chicken
Source: PLoS One. 2023 Aug 22;18(8):e0290436. doi: 10.1371/journal.pone.0290436 (PMC10443837; doi:10.1371/journal.pone.0290436)
Supplement: S3 Fig — a: Our designed primer location in the candidate sequence of Okinawa rail MDA5 from the draft genome. b-d: Designed primers of this study. (PDF) [file pone.0290436.s003.pdf]

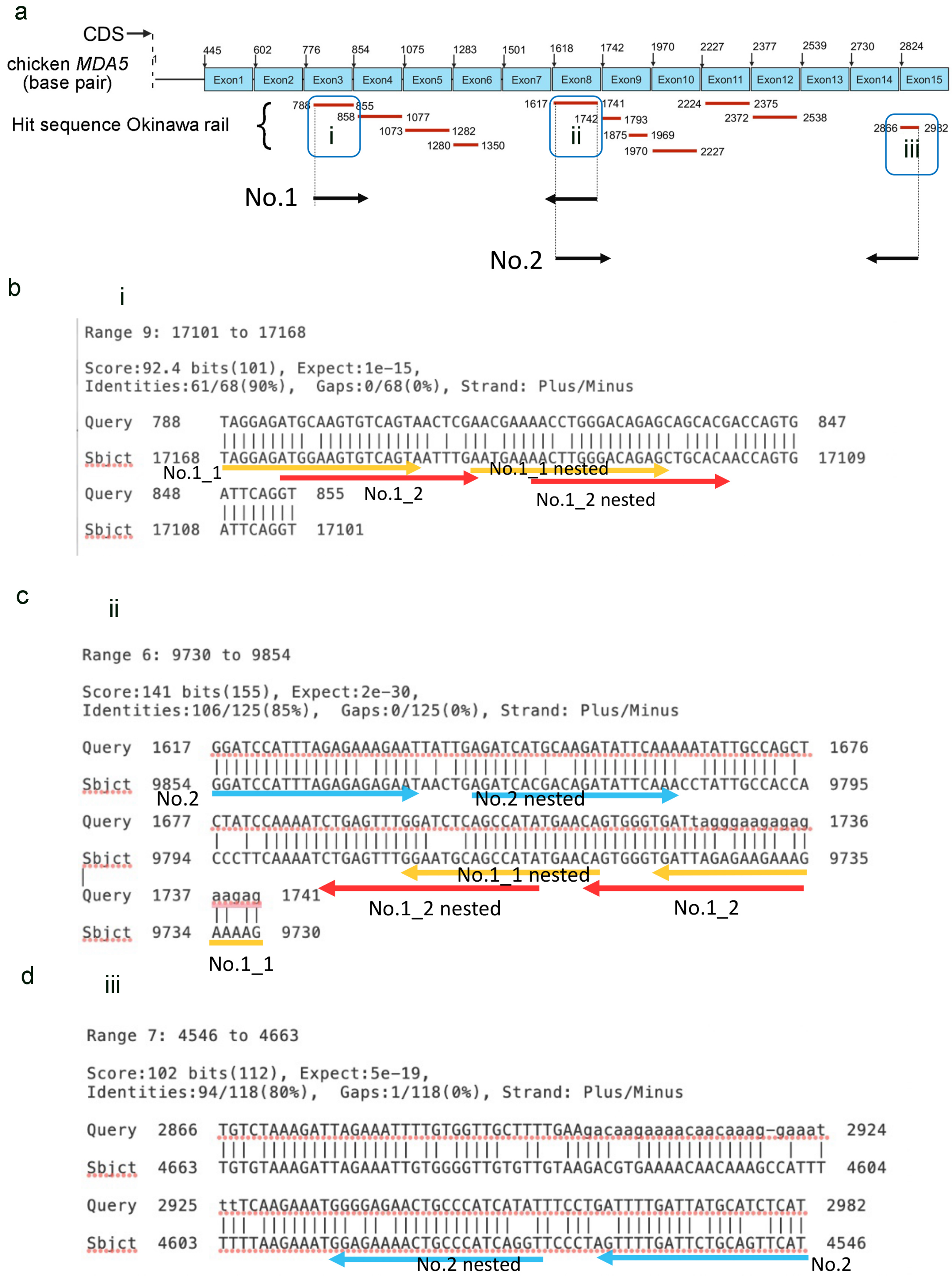
a
CDS
chicken MDA5
(base pair)
445 602 776 854 1075 1283 1501 1618 1742 1970 2227 2377 2539 2730 2824
Exon1 Exon2 Exon3 Exon4 Exon5 Exon6 Exon7 Exon8 Exon9 Exon10 Exon11 Exon12 Exon13 Exon14 Exon15
Hit sequence Okinawa rail
788 855
858 1077
1073 1282
1280 1350
1617 1741
1742 1793
1875 1969
1970 2227
2224 2375
2372 2538
2866 2982
i
ii
iii
No.1
No.2
b
i
Range 9: 17101 to 17168
Score:92.4 bits(101), Expect:1e-15,
Identities:61/68(90%), Gaps:0/68(0%), Strand: Plus/Minus
Query 788 TAGGAGATGCAAGTGTCAGTAACTCGAACGAAAACCTGGGACAGAGCAGCACGACCAGTG 847
Sbjct 17168 TAGGAGATGGAAGTGTCAGTAATTTGAATGAAAACTTGGGACAGAGCTGCACAACCAGTG 17109
No.1_1
No.1_1 nested
Query 848 ATTCAGGT 855
No.1_2
No.1_2 nested
Sbjct 17108 ATTCAGGT 17101
c
ii
Range 6: 9730 to 9854
Score:141 bits(155), Expect:2e-30,
Identities:106/125(85%), Gaps:0/125(0%), Strand: Plus/Minus
Query 1617 GGATCCATTTAGAGAAAGAATTATTGAGATCATGCAAGATATTCAAAAATATTGCCAGCT 1676
Sbjct 9854 GGATCCATTTAGAGAGAGAATAACTGAGATCACGACAGATATTCAAACCTATTGCCACCA 9795
No.2
No.2 nested
Query 1677 CTATCCAAAATCTGAGTTTGGATCTCAGCCATATGAACAGTGGGTGATtagggaagagag 1736
Sbjct 9794 CCCTTCAAAATCTGAGTTTGGAATGCAGCCATATGAACAGTGGGTGATTAGAGAAGAAAG 9735
No.1_1 nested
Query 1737 aagag 1741
No.1_2 nested
No.1_2
Sbjct 9734 AAAAG 9730
No.1_1
d
iii
Range 7: 4546 to 4663
Score:102 bits(112), Expect:5e-19,
Identities:94/118(80%), Gaps:1/118(0%), Strand: Plus/Minus
Query 2866 TGTCTAAAGATTAGAAATTTTGTGGTTGCTTTTGAAgacaagaaaacaacaaag-gaaat 2924
Sbjct 4663 TGTGTAAAGATTAGAAATTGTGGGGTTGTGTTGTAAGACGTGAAAACAACAAAGCCATTT 4604
Query 2925 ttTCAAGAAATGGGGAGAACTGCCCATCATATTTCCTGATTTTGATTATGCATCTCAT 2982
Sbjct 4603 TTTTAAGAAATGGAGAAAACTGCCCATCAGGTTCCCTAGTTTTGATTCTGCAGTTCAT 4546
No.2 nested
No.2
