# Supplementary material for: Cultured fibroblasts of the Okinawa rail present delayed innate immune response compared to that of chicken
Source: PLoS One. 2023 Aug 22;18(8):e0290436. doi: 10.1371/journal.pone.0290436 (PMC10443837; doi:10.1371/journal.pone.0290436)

a

b

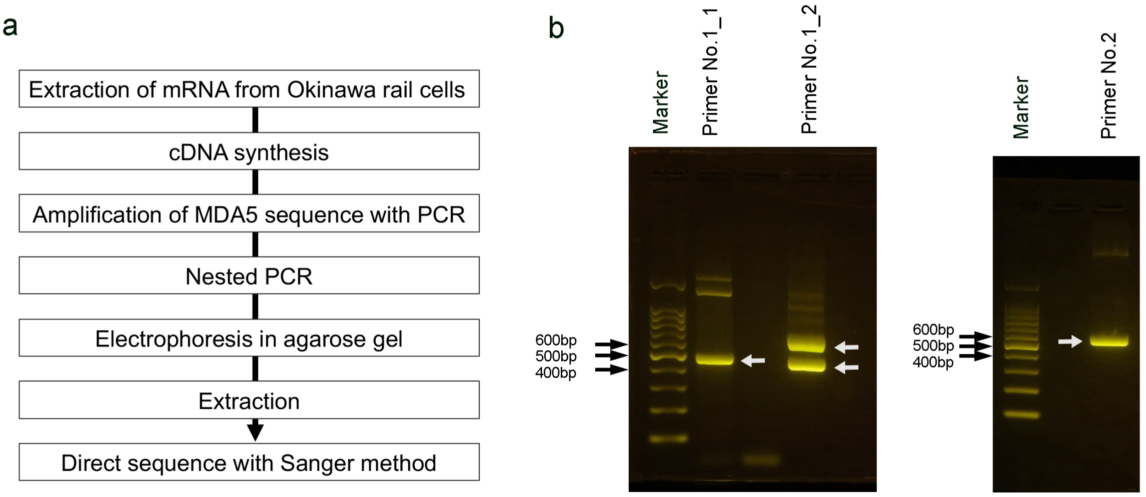

c

MKKLGQSCTTSDSDEDGEQSFT*ARSDPE*LPDGSCKASTEWDNIIICLPTGSGKPEWLFTLPKITWIRRKE
HQSL*LLYLLNKIPLV*EHFQKEFNQFLKHQYQVIGLRGDSQLKI*FPEVVRMMLASSLSTYP**VIVKRNQKR*
RCPVIRGLNYM*HGIPASVKWRLLSQLHFTSCLPSLPRAEVTV*PFQKLRITSRHTSLLDPFRERITEITTDIQT
YCHHPSKSEFGMQPYEQWVIREERKARS*VQDDESTYAVVALSGSGAVEHEDVNIFFVKMMYRAHQQVQ
KMRQD*YLNKVFITALSFPFKEDLYCNKYFLFEFYLGKESFE*YYLLHMTLNPFANIYFIIYVFWAFRKVCFSIG
KRGNTQVRLSGNCVKIRNCGVVL*DGKQQSHFFKKWRKSPIIGV*

d

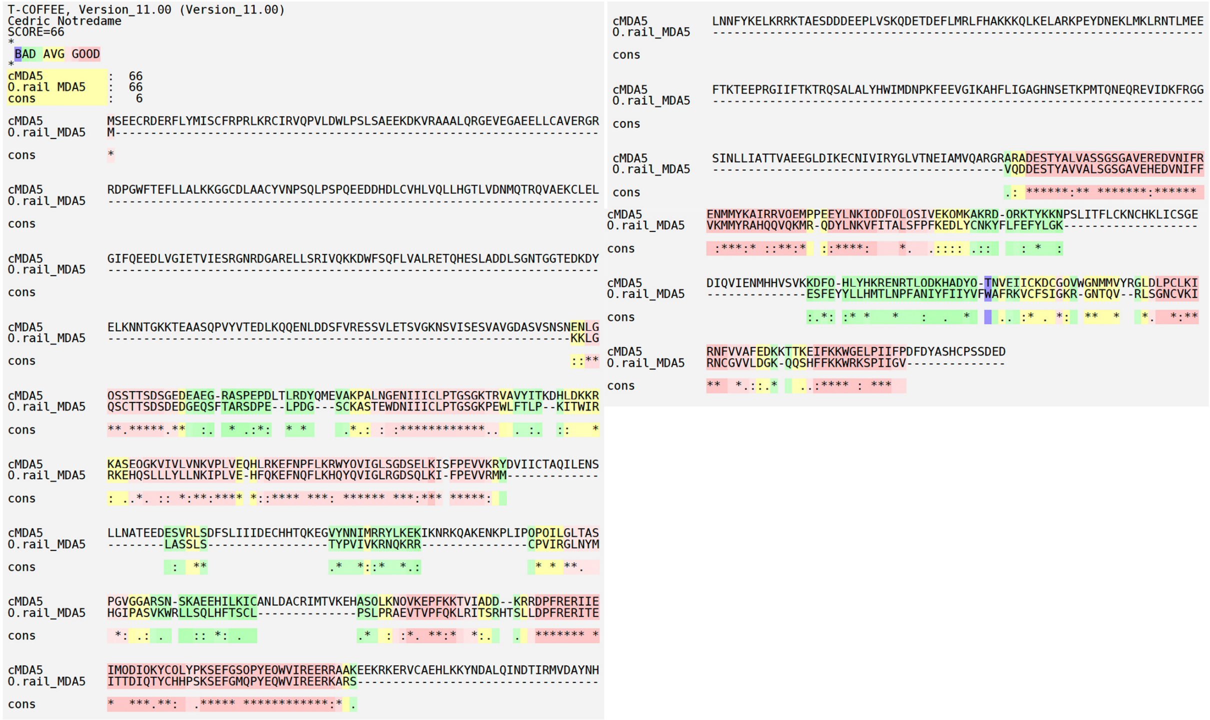

Supplement: S4 Fig — a: Flow of obtaining the Okinawa rail MDA5. b: Image of electrophoresis after nested PCR. We extracted the amplification sequence from the white arrows. c: Amino acids sequence of our obtained Okinawa rail MDA5. Those sequences were translated from our obtained Okinawa rail MDA5 sequence (Fig 6A). Red asterisks are stop codons. d: Homology between chicken MDA5 and our Okinawa rail MDA5 amino acids sequence. (PDF) [file pone.0290436.s004.pdf]
