# Supplementary material for: Cultured fibroblasts of the Okinawa rail present delayed innate immune response compared to that of chicken
Source: PLoS One. 2023 Aug 22;18(8):e0290436. doi: 10.1371/journal.pone.0290436 (PMC10443837; doi:10.1371/journal.pone.0290436)
Supplement: S5 Fig — The orange line is the 81bp cDNA fragment of Fig 4H. The blue highlight sequences are translated amino acids sequences of Okinawa rail that of 81bp cDNA fragment. The green highlight sequences are translated amino acids sequences of chicken that of 81bp cDNA fragment. Asterisks are stop codons. (PDF) [file pone.0290436.s005.pdf]

Gallus gallus melanoma differentiation-associated protein 5 mRNA, complete cds

Sequence ID: **GU570144.1** Length: 3006 Number of Matches: 2

Range 1: 818 to 1283

Score	Expect	Identities	Gaps	Strand	Frame
471 bits(521)	3e-130()	390/468(83%)	15/468(3%)	Plus/Plus	

Okinawa rail → CDS: Putative 1 / Query

Chicken → Sbjct / CDS:melanoma differe

```
CDS: Putative 1     1      K  L  G  Q  S  C  T  T  S  D  S  D  E  D        G  E  Q  S
Query               11    AAAAACTGGGACAGAGCTGCACAACCAGTGATTCAGATGAAGATG-----GAGAGCAGAG  65
Sbjct               818   AAAACCTGGGACAGAGCAGCACGACCAGTGATTCAGGTGAAGATGAAGCAGAGGGCAGAG  877
CDS:melanoma differe 273  E  N  L  G  Q  S  S  T  T  S  D  S  G  E  D  E  A  E  G  R

CDS: Putative 1     19      F  T  *  A  R  S  D  P  E  *  L  P  D  G  S  C  K  A  S  T
Query               66    CTTCACCTGAGCCAGATCTGATCCTGAGTGATTGCCAGATGGAAGTTGCAAAGCCAGCAC  125
Sbjct               878   CTTCACCTGAGCCAGATCTCACCCTGAGAGATTACCAGATGGAAGTTGCAAAACCAGCAC  937
CDS:melanoma differe 293  A  S  P  E  P  D  L  T  L  R  D  Y  Q  M  E  V  A  K  P  A

CDS: Putative 1     37      E  W  D  N  I  I  I  C  L  P  T  G  S  G  K  P  E  W  L
Query               126   TGAATGGG-ATAACATTATAATATGTCTCCCTACAGGCAGTGGTAA-ACCAGAGTGGCTA  183
Sbjct               938   TGAATGGGGAGAATATTATAATATGTCTCCCTACAGGCAGTGGCAAGACCAGAGTGGCTG  997
CDS:melanoma differe 313  L  N  G  E  N  I  I  I  C  L  P  T  G  S  G  K  T  R  V  A

CDS: Putative 1     56     F  T  L  P  K  I  T  W  I  R  R  K  E  H  Q  S  L  *     L
Query               184   TTTACATTACCAAAGATCACTTGGATAAGAAGAAAAGAGCATCAGAGTCTGTA---GTTA  240
Sbjct               998   TTTACATTACCAAAGATCACTTGGATAAGAAGAGAAAAGCATCAGAGCAAGGAAAAGTTA  1057
CDS:melanoma differe 333  V  Y  I  T  K  D  H  L  D  K  K  R  K  A  S  E  Q  G  K  V

CDS: Putative 1     74      L  Y  L  L  N  K  I  P  L  V  *  E  H  F  Q  K  E  F  N  Q
Query               241   CTATACTTGTTAAATAAGATACCACTGGTATAAGAGCATTTCCAAAAGGAGTTTAATCAA  300
Sbjct               1058  TAGTACTTGTTAA-TAAGGTACCGTTAGTGGAACAGCATTTACGAAAGGAGTTTAATCCA  1116
CDS:melanoma differe 353  I  V  L  V  N  K  V  P  L  V  E  Q  H  L  R  K  E  F  N  P

CDS: Putative 1     93      F  L  K  H  Q  Y  Q  V  I  G  L  R  G  D  S  Q  L  K  I  *
Query               301   TTCCTGAAGCATCAGTATCAGGTTATTGGTTTAAGGGGTGATTCCCAACTGAAAATCTGA  360
Sbjct               1117  TTCCTGAAACACTGGTATCAAGTTATTGGCTTAAGTGGTGATTCTGAGCTGAAAATCTCG  1176
CDS:melanoma differe 373  F  L  K  H  W  Y  Q  V  I  G  L  S  G  D  S  E  L  K  I  S

CDS: Putative 1     112     F  P  E  V  V     R  M  M  L  A  S  S  L     S  T  Y  P  *  *
Query               361   TTTCCTGAAGTTGTC--AGGATGATGTTGGCATCATCCTTA-AGCACATATCCTTGATAA  417
Sbjct               1177  TTTCCTGAAGTTGTCAAAGGAT-ATGATGTCATCATCTGTACAGCACAGATCCTGGAGAA  1235
CDS:melanoma differe 393  F  P  E  V  V  K  G     Y  D  V  I  I  C  T  A  Q  I  L  E  N

CDS: Putative 1     129     V  I  V  K  R  N  Q  K  R  *  R  C  P  V  I  R
Query               418   GTCATTGTTAAACGCAACCAAAAAAGATGAAGGTGTCCAGTTATCAGA  465
Sbjct               1236  TTCACTGCTAAATGCAACTGAAGAAGATGAAAGTGTCCGCTTGTCAGA  1283
CDS:melanoma differe 413    S  L  L  N  A  T  E  E  D  E  S  V  R  L  S  D
```
