# Supplementary material for: Cultured fibroblasts of the Okinawa rail present delayed innate immune response compared to that of chicken
Source: PLoS One. 2023 Aug 22;18(8):e0290436. doi: 10.1371/journal.pone.0290436 (PMC10443837; doi:10.1371/journal.pone.0290436)
Supplement: S1 Table — (PDF) [file pone.0290436.s006.pdf]

Species	Gene name		Sequence (5' to 3')	Length (bp)
Chicken	*GAPDH*	Forward	GAGGCCAGTTCTGTTCCCTT	88
		Reverse	ATCAGTTTCTATCAGCCTCTCCC	
	MDA5	Forward	CTCCCTACAGGCAGTGGCAAA	132
		Reverse	ATGCTGTTCCACTAACGGTACCTT	
	LGP2	Forward	AGCCCACGAAGCAGTACGACCT	143
		Reverse	CTGCTCATACACCTGCGTGCCGAA	
	IL6	Forward	AGAAATGCCTGACGAAGCTCTCC	99
		Reverse	ACTCGACGTTCTGCTTTTCGCTA	
	IL1β	Forward	TACACCCGCTCACAGTCCT	132
		Reverse	CGCAATGTTGAGCCTCACT	
	IFNβ	Forward	CCTTCAGAATACGGCTCCACCT	108
		Reverse	ATGGCTGCTTGCTTCTTGTCC	
	Mx1	Forward	ATATGACAGAAAGATCCGACCT	105
		Reverse	CGGTCTCCAATCACTGCAA	
	TLR3	Forward	CTTTCGCACATTAACAGGT	126
		Reverse	CTCTAATTTGACAGCCACT	
