# Supplementary material for: Cultured fibroblasts of the Okinawa rail present delayed innate immune response compared to that of chicken
Source: PLoS One. 2023 Aug 22;18(8):e0290436. doi: 10.1371/journal.pone.0290436 (PMC10443837; doi:10.1371/journal.pone.0290436)
Supplement: S2 Table — (PDF) [file pone.0290436.s007.pdf]

Species	Gene name		Sequence (5' to 3')	Length (bp)
Okinawa rail	*GAPDH*	Forward	TTATCATCTCTGCCCCCTC	82
		Reverse	ATTTTCAGAGACTTGTCATACTTG	
	RIG-I	Forward	ACTGTCACCGAAACAATAGAGC	80
		Reverse	TTCTCTCTAACAGTGGATATGGC	
	MDA5	Forward	GTTGCAAAGCCAGCACT	82
		Reverse	AATGTAAATAGCCACTCTGGT	
	LGP2	Forward	GTGATAGATGAGTGCCACCACACG	89
		Reverse	TGCTGCCCGCTGAGCTT	
	IL6	Forward	GAAGTTTACCGTCTGCGCGAACAGC	82
		Reverse	CCCGTCTTCCTCCGTCACCTT	
	IL1β	Forward	TAGGAAGCCGACATCAGGAGAGAC	97
		Reverse	CAAAGCGAGTGGTGCCCTC	
	IFNβ	Forward	AAACCCTCCATCACCTCTCTGAC	150
		Reverse	TCTTGGAGAGCGTCCTGTTGTCTGG	
	Mx1	Forward	AAAGATCCGACCCTGCAT	123
		Reverse	TCTAGGACGGAGCTTTTCCCA	
	TLR3	Forward	AATCCAGAACCTCTCGCTGA	150
		Reverse	CTTGGAAGCCACTGAAACGA	
