# Supplementary material for: Cultured fibroblasts of the Okinawa rail present delayed innate immune response compared to that of chicken
Source: PLoS One. 2023 Aug 22;18(8):e0290436. doi: 10.1371/journal.pone.0290436 (PMC10443837; doi:10.1371/journal.pone.0290436)
Supplement: S3 Table — (PDF) [file pone.0290436.s008.pdf]

Species	Gene name		Sequence (5' to 3')	Length (bp)
Domestic duck	*GAPDH*	Forward	GCTGGCATTGCACTGAACGACCAT	87
		Reverse	CAAGTCCACCACACGGTTGCT	
	RIG-I	Forward	AACACTACAGGCAACCACCCTT	93
		Reverse	ACCTAAAATCTGTGGCAGCTGAC	
	MDA5	Forward	ATCTCAGCCATATGAACAGTGGGT	80
		Reverse	CACAGACACGTTCCTTGCGTTT	
	LGP2	Forward	ACAGCAACCAGACCAGGCACA	148
		Reverse	CCGTAGCGCACCACGATGTTGCAC	
	IL6	Forward	TCTCCAGCGGGCTTTTCACCT	122
		Reverse	CCTTATCGTCGTTGCCAGATGCTT	
	IL1β	Forward	CCCGTCACCTTCCAGAGGCTTG	115
		Reverse	AATCCAGCATGAAGCACTTGTGGT	
