# Supplementary material for: Cultured fibroblasts of the Okinawa rail present delayed innate immune response compared to that of chicken
Source: PLoS One. 2023 Aug 22;18(8):e0290436. doi: 10.1371/journal.pone.0290436 (PMC10443837; doi:10.1371/journal.pone.0290436)
Supplement: S4 Table — (PDF) [file pone.0290436.s009.pdf]

Species	Gene name		Sequence (5' to 3')	Length (bp)
Whooper Swan	*GAPDH*	Forward	GTCCATGCTATCACAGCCACAC	128
		Reverse	ACTTTTCCCACAGCCTTAGCAG	
	RIG-I	Forward	AACAATGTTAGAAGTCGACCC	81
		Reverse	TGAATCTCTTCGCACTCCC	
	MDA5	Forward	CACTATCCGAATGGTCGATG	112
		Reverse	CTGTTTTGATACTGCTGGTT	
	LGP2	Forward	CGCAAGTACAACGACGCTTTGCTGA	110
		Reverse	TCGGTGGGGTCCTTCATGTCCCT	
	IL6	Forward	CTCACAAAGCTGAAGTCGGAT	113
		Reverse	TTCAAATAGCGAACAGCCCTC	
	IL1β	Forward	CTTCCCCGGCTGGTTCGTCT	105
		Reverse	CGCCCGCTCAGCTTGTAGGT	
