# Supplementary material for: Cultured fibroblasts of the Okinawa rail present delayed innate immune response compared to that of chicken
Source: PLoS One. 2023 Aug 22;18(8):e0290436. doi: 10.1371/journal.pone.0290436 (PMC10443837; doi:10.1371/journal.pone.0290436)
Supplement: S5 Table — (PDF) [file pone.0290436.s010.pdf]

Species	Gene name		Sequence (5' to 3')	Length (bp)
Chicken and Okinawa rial	*GAPDH*	Forward	TTACTGGAATGGCTTTCCGTGTG	113
		Reverse	AGCAGCCTTCACTACCCTC	
		Probe	[FAM]TTCTCCAGACGGCAGGTCAGG[BHQ1]	
	MDA5	Forward	CTTCACCTGAGCCAGAT	148
		Reverse	TCTTATCCAAGTGATCTTTGGTA	
		Probe	[FAM]TTATAATATGTCTCCCTACAG[BHQ1]	
