# Supplementary material for: Cultured fibroblasts of the Okinawa rail present delayed innate immune response compared to that of chicken
Source: PLoS One. 2023 Aug 22;18(8):e0290436. doi: 10.1371/journal.pone.0290436 (PMC10443837; doi:10.1371/journal.pone.0290436)
Supplement: S6 Table — (PDF) [file pone.0290436.s011.pdf]

Species	Primer name		Sequence (5' to 3')
Okinawa rai	No.1_1	Forward	TAGGAGATGGAAGTGTCAGTA
		Reverse	CTTTTCTTTCTTCTCTAATCA
	No.1_1nested	Forward	AATGAAAACTTGGGACAGAGC
		Reverse	TGTTCATATGGCTGCATTCCA
	No.1_2	Forward	ATGGAAGTGTCAGTAATTTGA
		Reverse	TTTCTTCTCTAATCACCCACT
	No.1_2 nested	Forward	AACTTGGGACAGAGCTGCACA
		Reverse	TATGGCTGCATTCCAAACTCA
	No.2	Forward	GGATCCATTTAGAGAGAGAAT
		Reverse	ATGAACTGCAGAATCAAAACT
	No.2 nested	Forward	AGATCACGACAGATATTCAAA
		Reverse	ACCTGATGGGCAGTTTTCTCC
