# Supplementary material for: Cultured fibroblasts of the Okinawa rail present delayed innate immune response compared to that of chicken
Source: PLoS One. 2023 Aug 22;18(8):e0290436. doi: 10.1371/journal.pone.0290436 (PMC10443837; doi:10.1371/journal.pone.0290436)
Supplement: S7 Table — (PDF) [file pone.0290436.s012.pdf]

Fig number	Gene name		normal distribution
Fig.3a	MDA5	0μg/mL	Yes
		5μg/mL	Yes
		50μg/mL	Yes
	LGP2	0μg/mL	Yes
		5μg/mL	Yes
		50μg/mL	Yes
Fig.3b	RIG-1	0μg/mL	Yes
		5μg/mL	Yes
		50μg/mL	Yes
	MDA5	0μg/mL	Yes
		5μg/mL	Yes
		50μg/mL	Yes
	LGP2	0μg/mL	Yes
		5μg/mL	Yes
		50μg/mL	Yes
Fig.5c	Chicken	0μg/mL	Yes
		5μg/mL	Yes
		50μg/mL	Yes
	OKinawa rail	0μg/mL	No
		5μg/mL	Yes
		50μg/mL	Yes
Fig.7a	IL6	0μg/mL	Yes
		5μg/mL	Yes
		50μg/mL	No
	IL1 beta	0μg/mL	Yes
		5μg/mL	Yes
		50μg/mL	Yes
Fig.7b	IL6	0μg/mL	Yes
		5μg/mL	Yes
		50μg/mL	Yes
	IL1 beta	0μg/mL	Yes
		5μg/mL	Yes
		50μg/mL	Yes
Fig.8a	Chicken	0μg/mL	Yes
		5μg/mL	Yes
		50μg/mL	Yes
	Okinawa rail	0μg/mL	Yes
		5μg/mL	Yes
		50μg/mL	Yes
Fig.8b	Chicken	0μg/mL	Yes
		5μg/mL	Yes
		50μg/mL	Yes
	Okinawa rail	0μg/mL	Yes
		5μg/mL	Yes
		50μg/mL	Yes

Fig number	Gene name		normal distribution
Fig.8c	Chicken	0μg/mL	Yes
		5μg/mL	Yes
		50μg/mL	Yes
	Okinawa rail	0μg/mL	Yes
		5μg/mL	Yes
		50μg/mL	Yes
Fig.8d	Chicken	0μg/mL	Yes
		5μg/mL	Yes
		50μg/mL	No
	Okinawa rail	0μg/mL	Yes
		5μg/mL	Yes
		50μg/mL	No
Fig.9c	Live cell	0μg/mL	Yes
		50μg/mL	Yes
	Early apototic	0μg/mL	Yes
		50μg/mL	No
	Late Apop/Dead	0μg/mL	Yes
		50μg/mL	Yes
Fig.9d	Live cell	0μg/mL	Yes
		50μg/mL	Yes
	Early apototic	0μg/mL	Yes
		50μg/mL	Yes
	Late Apop/Dead	0μg/mL	No
		50μg/mL	Yes
Fig.10a	Chicken	0μg/mL	Yes
		5μg/mL	Yes
		50μg/mL	Yes
	Okinawa rail	0μg/mL	Yes
		5μg/mL	Yes
		50μg/mL	Yes
Fig.10b	Chicken	0μg/mL	Yes
		5μg/mL	Yes
		50μg/mL	Yes
	Okinawa rail	0μg/mL	Yes
		5μg/mL	Yes
		50μg/mL	Yes
S3 Fig.	Chicken	0μg/mL	Yes
		5μg/mL	Yes
		50μg/mL	Yes
	Okinawa rail	0μg/mL	Yes
		5μg/mL	Yes
		50μg/mL	No
